# The Quinoline Photoremovable Group (PPG) Platform—A Medicinal Chemist's Approach for Photocage Development and Applications

**DOI:** 10.1002/med.22111

**Published:** 2025-04-12

**Authors:** Bence Kontra, Zoltán Mucsi, Janez Ilaš, Petra Dunkel

**Affiliations:** ^1^ Institute of Organic Chemistry Semmelweis University Budapest Hungary; ^2^ Department of Biological Chemistry BrainVision Center Budapest Hungary; ^3^ Department of Chemistry Femtonics Ltd. Budapest Hungary; ^4^ Institute of Chemistry University of Miskolc Miskolc Hungary; ^5^ Faculty of Pharmacy University of Ljubljana Ljubljana Slovenia; ^6^ Center for Pharmacology and Drug Research & Development Semmelweis University Budapest Hungary

**Keywords:** drug delivery systems, neuronal signaling, photoremovable protecting groups, quinoline photocages, structure‐property relationships, two‐photon absorption, two‐photon uncaging

## Abstract

Photoremovable protecting groups (PPGs) offer a straightforward solution for the temporary inactivation of biologically active substrates and their subsequent controlled release by light irradiation. Their relatively easy design and mode of application have made them useful tools for studying dynamic biological processes in vitro and in vivo. Recently, there has been a growing body of data investigating their potential application in the development of drug delivery systems. Of the various PPG scaffolds in use, quinoline photocages have a history of about 20 years. The structure‐property relationships of quinoline PPGs, as well as alternative multibranch designs based on quinoline monomers have been thoroughly studied both experimentally and theoretically. Therefore, quinoline PPGs serve as a representative study of PPG development, showing how the various applications of quinoline photocages followed the chemical optimization or how the applications drove the chemical design. Since the raison d’être of PPGs lies in their application for light‐activated release of various substrates or performing light‐activated structural changes in materials, it is crucial to understand how PPGs are selected and utilized by their end‐users, who are often not chemists themselves. Therefore, we discuss whether the conclusions drawn from the selected quinoline PPG family could lead to more general insights for the field as whole. As PPG‐related applications still rely heavily on a limited number of chemical scaffolds, it is worth considering, what could be the reasons for the slow uptake of novel chemical scaffolds.

**Abbreviations**
1P: one‐photon
1PA: one‐photon absorption
1PE: one‐photon excitation
2P: two‐photon
2PA: two‐photon absorption
2PE: two‐photon excitation
3PE: three‐photon excitation
3′‐PyHQ: (7‐hydroxy‐8‐(pyridin‐3‐yl)quinolin−2‐yl)methyl
5‐HT: 5‐hydroxytriptamine or serotonin
AMPA: α‐amino‐3‐hydroxy‐5‐methyl‐4‐isoxazolepropionic acid
BFP: blue fluorescent protein
Bhc: 6‐bromo‐7‐hydroxycoumarin‐4‐ylmethyl
BHQ: 8‐bromo‐7‐hydroxyquinoline‐2‐ylmethyl
BNSF: ((2,7‐bis‐(4‐nitro‐8‐[3‐(2‐propyl)‐styryl])‐9,9‐bis‐[1‐(3,6‐dioxaheptyl)]‐fluorene
BODIPY: boron‐dipyrromethene
cbl: chlorambucil
CHQ: 8‐chloro‐7‐hydroxyquinoline‐2‐ylmethyl
CLSM: confocal laser scanning microscopy
CyHQ: 8‐cyano‐7‐hydroxyquinoline‐2‐ylmethyl
DA: dopamine
DCT: charge transfer distance
DDS: drug delivery system
DFT: density functional theory
DMAPh: dimethylaminophenanthridine
DMAQ: dimethylaminoquinoline‐2‐ylmethyl
DMNB: 4,5‐dimethoxy‐4‐nitrobenzyl
DMNPB: 3‐(4,5‐dimethoxy‐2‐nitrophenyl)‐2‐butyl
DNA: deoxyribonucleic acid
DNI: 4‐methoxy‐5,7‐dinitroindolinyl
DOTAGA: 1,4,7,10‐tetraazacyclo‐dodecane‐1‐glutaric acid‐4,7,10‐triacetic acid
EANBP: 2‐(4′‐(bis((2‐methoxyethoxy)ethyl)amino)‐4‐nitro‐[1,1′‐biphenyl]‐3‐yl)propyl
EDG: electron‐donating
ESA: excited state absorption
ESPT: excited state proton transfer
EWG: electron‐withdrawing
GABA: gamma‐aminobutyric acid
GFP: green fluorescent protein
GIRK: G‐protein‐coupled inwardly rectifying potassium channel
Glu: glutamic acid
GM: Göppert‐Mayer (1 GM = 10^−50^ cm^4^ s photon^−1^)
GRAB_5‐HT_
: G‐protein‐coupled receptor (GPCR)‐activation‐based 5‐HT sensor
HOMO: highest occupied molecular orbital
HTO: hydroxylated thiazole orange
ICT: internal charge transfer
Ind: inducer
ISC: intersystem crossing
ISRE: intermediate state resonance enhancement
KA: kainic acid
KMOPS: 3‐(*N*‐morpholino)propanesulfonic acid buffer with KCl
LC‐MS: liquid chromatography–mass spectrometry
LED: light emitting diode
LUMO: lowest unoccupied molecular orbital
MCM: (7‐methoxycoumarin‐4‐yl)methyl
MNDQ: 5‐methoxy‐8‐nitro‐1,2‐dihydroquinolinyl
MNI: 4‐methoxy‐7‐nitroindolinyl
MO: morpholino oligonucleotide
mRNA: messenger RNA
MSN: mesoporous silica nanoparticle
MTT: 3‐(4,5‐dimethylthiazol−2‐yl)‐2,5‐diphenyltetrazolium bromide
NHQ: 8‐nitro‐7‐hydroxyquinoline‐2‐ylmethyl
NIR: near‐infrared
NMDAR: *N*‐methyl‐d‐aspartate receptor
NMR: nuclear magnetic resonance
PBS: phosphate buffered saline
PDT: photodynamic therapy
PEG: polyethylene glycol
PET: photoinduced electron transfer
PFPE: perfluorinated polyether
PPG: photoremovable protecting group
PS: potassium sorbate
RNA: ribonucleic acid
RuBi: ruthenium bipyridyl
SAR: structure–activity relationship
S_N_1: unimolecular nucleophilic substitution
S_N_Ar: nucleophilic aromatic substitution
SNS: sodium 2‐naphthalenesulfonate
TA: transient absorption
TD‐DFT: time‐dependent density‐functional theory
TEG: triethylene glycol
TICT: twisted intramolecular charge transfer
TMP‐CyHQ: ((8‐cyano‐7‐hydroxy‐4‐(3,4,5‐trimethoxyphenyl)‐quinolin‐2‐yl)methyl
TPEF: two‐photon excited fluorescence
TQ: 7‐mercaptoquinoline‐2‐ylmethyl
TR^2^
: transient resonance Raman
TR^3^
: time‐resolved resonance Raman
TRF: time‐resolved fluorescence
TRIR: time‐resolved infrared spectroscopy
TRP: transient receptor potential
TRPV1: transient receptor potential cation channel subfamily V member 1
UV: ultraviolet
VNA: *N*‐vanillylnonanoylamide

## 1 Introduction

**Figure 1:**
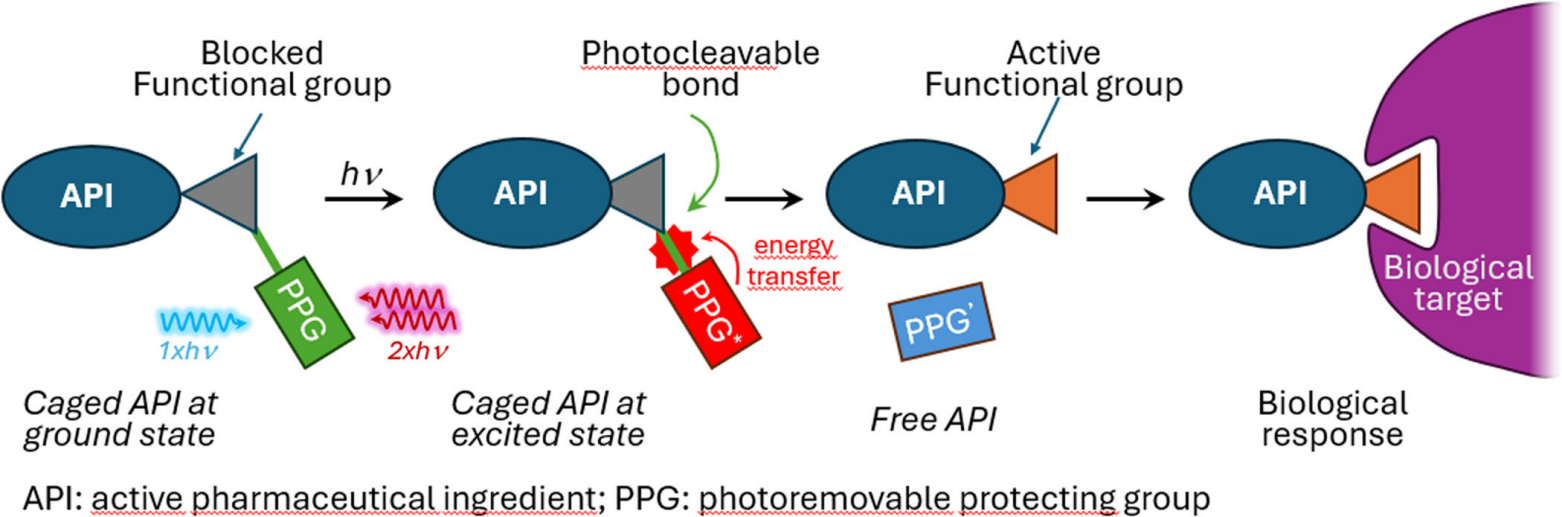
General and simplified concept of the uncaging mechanism. [Color figure can be viewed at wileyonlinelibrary.com]

Photoremovable protecting groups (PPGs) enable the temporary masking of the biological functions of bioactive ligands usually by forming a covalent bond to the functional group critical for the particular action, analogous to the “protection‐deprotection” sequence in synthetic organic chemistry [1, 2, 3, 4, 5]. PPGs possess a chromophore with appropriate ability for excitation to absorb energy and a chemical functionality with a photosensitive scissile bond (Figure 1). Biological activity can be restored by light irradiation at an appropriate wavelength, tuned to the PPG. The carefully selected chromophore of the PPG can be excited from the ground state to the excited state (S_0_ → S_1_) by a photon with specific wavelength. Later, the excited chromophore transitions to its triplet state (T_1_) and the excess energy is transferred to the photocleavable bond, which cleaves to yield the released active ingredient and the remaining part of the cage scaffold.

**Figure 2:**
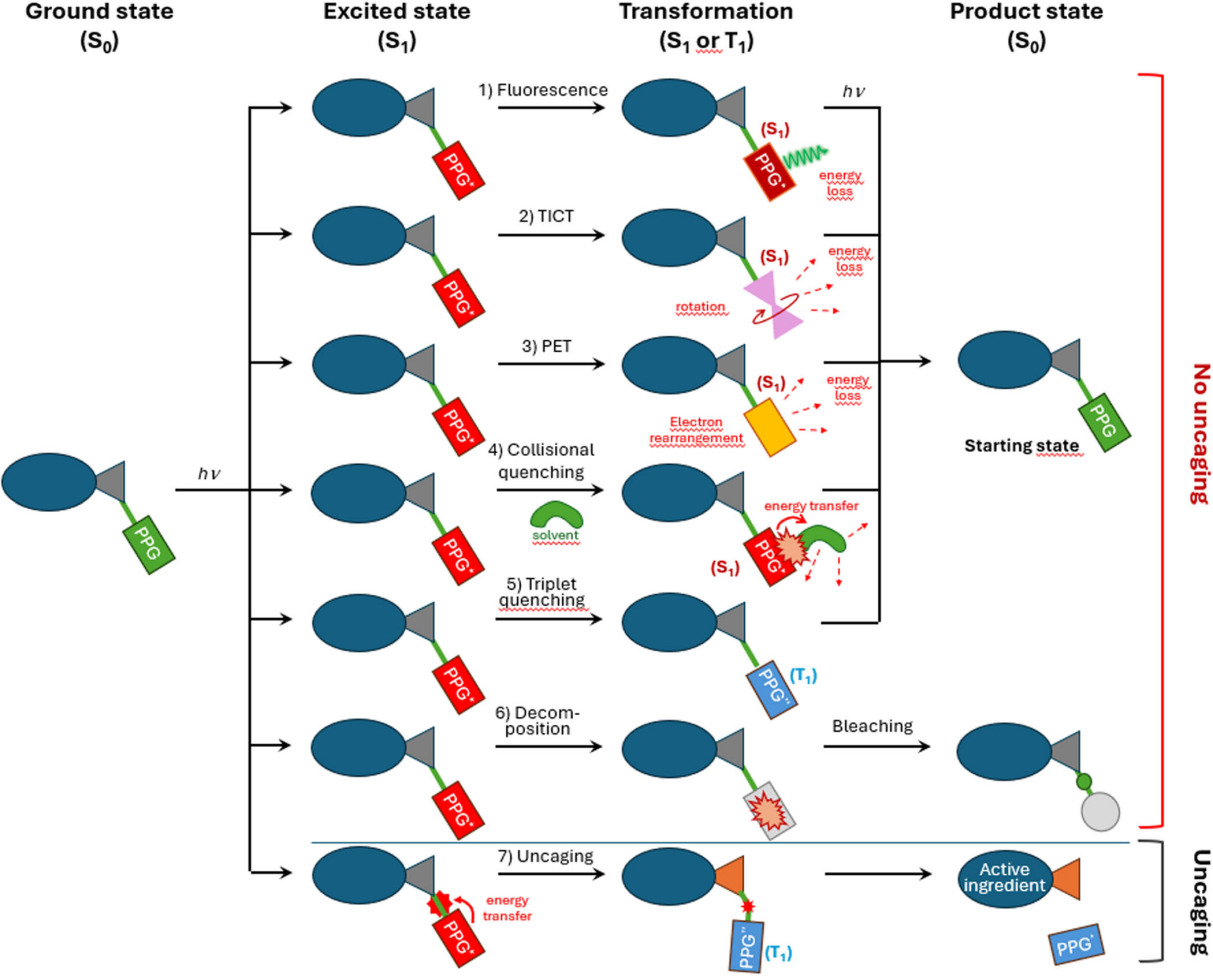
The most relevant photophysical processes after photoexcitation, including fluorescence, TICT, PET, collisional quenching, triplet quenching, photodecomposition and photocleavage/uncaging. [Color figure can be viewed at wileyonlinelibrary.com]

After entering the excited state (S_1_), the chromophore must bypass most of the typical quenching mechanisms and pass through the triplet state. Figure 2 lists some of the relevant concurrent and inefficient processes that should be avoided if an uncaging process is targeted. Interestingly, most of the basic PPG scaffolds are capable of emitting fluorescence [Figure 2: **1) fluorescence**] and losing energy, thus canceling the uncaging process. This fundamental property is one of the most important issues to be solved in the development of PPGs. Twisted intramolecular charge transfer (TICT) [Figure 2: **2) TICT**] refers to a photophysical process in which a molecule undergoes a rotation around a bond upon excitation, resulting in a distinct charge‐transfer state and dissipation of the excess energy. In photoinduced electron transfer (PET) [Figure 2: **3) PET**] the absorption of light triggers the transfer of an electron between a donor and an acceptor, which plays a key role in quenching and energy conversion. Collisional quenching [Figure 2: **4) Collisional quenching**] is the deactivation of the excited state through energy transfer during collisions with solvent molecules, without affecting the molecule's chemical structure. Triplet quenching [Figure 2: **5) Triplet quenching**] is a process in which the excited triplet state (T_1_) of a molecule can dissipate the excess of energy (e.g., through interaction with oxygen), thereby preventing photochemical reactions. Bleaching refers to the irreversible loss of absorbance in a molecule, caused by non‐specific photochemical damage or the breakdown of the fluorophore's structure. The most common process of the uncaging mechanism [Figure 2: **7) Uncaging**] involves a transition from the S_1_ state to the T_1_ state, with subsequent bond breaking from the T_1_ state.

**Figure 3:**
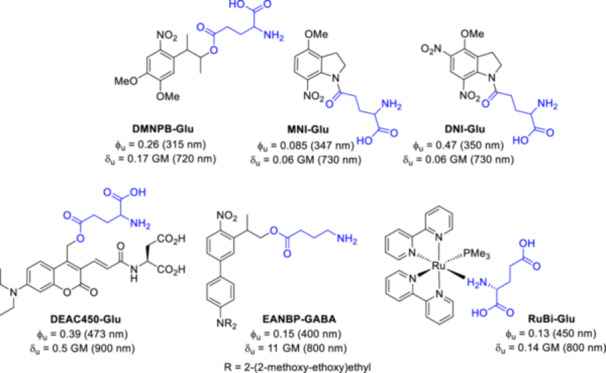
Selected examples of 2PE PPGs designed for the release of glutamate and GABA neurotransmitters. with some published parameters such as ϕ_u_ = 1P uncaging quantum yield, δ_u_ = 2P uncaging action cross section. DMNPB‐Glu—3‐(4,5‐dimethoxy‐2‐nitrophenyl)‐2‐butyl glutamate [11]; MNI‐Glu—4‐methoxy‐7‐nitroindolinyl glutamate [12, 13]; DNI‐Glu—4‐methoxy‐5,7‐dinitroindolinyl glutamate [14, 15, 16]; 7‐diethylaminocoumarin derivative DEAC450‐Glu [17]; EANBP‐GABA—2‐(4’‐(bis((2‐methoxyethoxy)ethyl)amino)‐4‐nitro‐[1,1’‐biphenyl]‐3‐yl)propyl GABA [18]; RuBi‐Glu—ruthenium bipyridyl glutamate [19]. [Color figure can be viewed at wileyonlinelibrary.com]

Photorelease by external light trigger allows fine spatial and temporal control of biological activity in the picosecond range and within a sub μm^3^ volume, that can be used to study dynamic biological processes [6, 7], or in clinical settings to optimize drugs with minimized unwanted off‐target toxicity [8, 9]. As with any activation method that relies on the use of external stimuli, light irradiation should not damage the cells (ideally λ > 400 nm light irradiation is used) and it should be able to penetrate some tissue layers (far red wavelengths). In this regard, one of the most promising approaches is to exploit the non‐linear optical phenomenon of two‐photon or three‐photon excitation (2PE or 3PE) [9]. In the 2PE process, the simultaneous absorption of two half‐energy photons leads to an excitation analogous to classical one‐photon excitation, by longer—red (λ > 600 nm) or near‐infrared (NIR; λ > 800 nm)—wavelengths. The wavelengths used for 2PE are already within the so‐called biological window for interventions. The field of 2PE has made significant progress recently, driven by the wide availability of Ti:Sapphire lasers and two‐photon microscopy techniques. The development of efficient 2PE PPGs remains challenging in many respects, however, several 2PE PPGs have become available in recent decades for various applications (Figure 3) [6, 7, 9, 10]. The selection of PPGs could be challenging, justifying the current chemical development efforts. PPGs must have an orthogonal excitation band as the active ingredient and other biological components, the difference should be at least 100 nm. PPGs must be non‐reactive and relatively stable in biological environments and should have good solubility. The caged molecule should be inactive on the desired target and all other receptors or enzymes. The residual cage scaffold should have low toxicity.

For a more comprehensive discussion of PPGs (“photocages”) in general [1, 20, 21, 22, 23] (Figure 3 shows some well‐established ones), two‐photon uncaging and design principles for two‐photon PPGs [3, 10, 24, 25, 26, 27], and some of the major applications of these probes, we refer the reader to the relevant earlier review articles [28, 29]. The aim of this review is to present a detailed analysis of a particular PPG family and to reflect on the broader implications that can be derived for the design and development of PPGs, and their uptake in the scientific community. The PPG family in question—2‐hydroxymethylquinoline‐based PPGs—was first described some 20 years ago, but despite the considerable amount of literature data, the quinoline PPG platform has not yet been reviewed in such detail.

**Figure 4:**
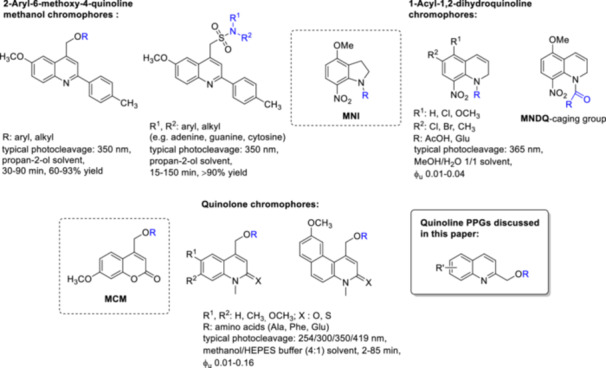
Miscellaneous quinoline PPGs. ϕ_u_ = 1P uncaging quantum yield. [Color figure can be viewed at wileyonlinelibrary.com]

Quinoline is one of the most important chemical scaffolds in the family of fluorescent probes and fluorescent chemosensors [30, 31, 32, 33, 34]; the effects of various structural changes or substitution patterns on the photophysical properties of different quinoline chromophores have been thoroughly investigated by experimental and theoretical methods [35]. Quinoline derivatives have played a pioneering and key role in the understanding of quinine receptors and have contributed to the development of anti‐inflammatory and antihypertensive drugs. Historically, quinine and quinoline compounds were among the first in which strong fluorescence was observed, marking a turning point in the field of photophysics and enabling advances in fluorescence spectroscopy. The fluorescence properties of quinoline‐based molecules have also facilitated research into receptor‐related signaling pathways, offering insights into their biological functions and therapeutic potential. Quinoline is also a privileged scaffold in medicinal chemistry, as a considerable number of natural products and drug molecules have this heterocyclic moiety in their structure [36, 37, 38]. In addition, among its different occurrences and fluorescence applications, several reports have described PPGs with different quinoline scaffolds. 2‐Aryl‐6‐methoxy‐4‐quinoline methanol chromophores have been effectively used as PPGs for amines or alcohols in a synthetic context via an ether or sulfonamide linkage with the substrate [39, 40, 41]. Quinolones with structural similarity to coumarins (i.e., 4‐hydroxymethyl‐1‐methylquinolin‐2(1*H*)‐ones and their thionated and benzo‐fused analogues) have also been described as useful PPGs for the carboxylic acid function of amino acids [42, 43, 44]. As a structural analogue of the widely used and commercially available 4‐methoxy‐7‐nitroindolinyl (MNI) and 4‐methoxy‐5,7‐dinitroindolinyl (DNI) PPGs [7], the 5‐methoxy‐8‐nitro‐1,2‐dihydroquinolinyl (MNDQ) chromophore was investigated for the caging of amino acids via an amide bond with a carboxylic acid function. *L*‐Glutamate released from MNDQ‐Glu at 365 nm or under 2PE photolysis at 720 nm induced a transient current at single dendritic spines with similar kinetics as the reference MNI‐Glu (Figure 4) [45]. Other substitution patterns of MNDQ were also investigated, but without testing the novel chromophores for biologically relevant substrates [46].

## 2 The Discovery, Substrate Scope, and Photocleavage Mechanism of 2‐Hydroxymethylquinoline PPGs

**Figure 5:**
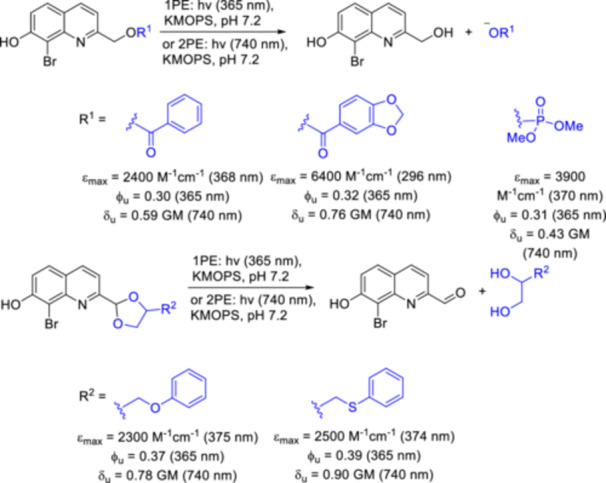
The substrate scope of BHQ. ε = 1P absorption, ϕ_u_ = 1P uncaging quantum yield, δ_u_ = 2P uncaging action cross section. [Color figure can be viewed at wileyonlinelibrary.com]

The application of 2‐hydroxymethylquinolines—on which the present work will focus—as PPGs was first reported in 2002 [47]. In this first report, the preparation and characterization of 8‐bromo‐7‐hydroxyquinoline‐2‐ylmethyl acetyl ester (BHQ‐OAc) was disclosed. The versatile preparation of 2‐hydroxymethylquinoline derivatives reported in the literature is usually based on the key steps of the synthetic pathway developed by Dore's group: Skraup or Doebner‐Miller synthesis starting from appropriately substituted aniline derivatives, Riley‐oxidation of the 2‐methyl group followed by NaBH_4_ reduction and introduction of the substrate via an esterification/alkylation/carbamate formation step. In direct comparison with the literature reference compounds 6‐bromo‐7‐hydroxycoumarin‐4‐ylmethyl acetate (Bhc‐OAc) and 4,5‐dimethoxy‐4‐nitrobenzyl acetate (DMNB‐OAc), BHQ‐OAc was found to be superior in terms of 1PE uncaging quantum efficiencies. In addition, BHQ‐OAc had a physiologically useful 2PE uncaging cross‐section. Other promising properties of BHQ‐OAc were its reasonable hydrolytic stability in the dark, its adequate water solubility, and its low fluorescence at 365 nm, which also enables its use in conjunction with fluorescent probes. The substrate scope of the BHQ protecting group has been demonstrated for various functionalities beyond carboxylic acids (Figure 5) [48]. Under simulated physiological conditions (KMOPS buffer, pH 7.2) the 1PE quantum efficiencies of BHQ‐protected carboxylates, phosphates and diols at 365 nm were in the range of 0.30–0.39 (corresponding to 60%–70% chemical yield), whereas the 2P uncaging action cross sections ranged from 0.43 to 0.90 GM, depending on the substrate. As another important addition to the substrate scope, the efficient caging and photorelease of cysteine thiol functionalities in the context of native chemical ligation was recently demonstrated [49]. Further additions to the substrate scope of BHQ‐OAc and its analogues are discussed in the following sections, with a focus on pharmacologically relevant examples. The other 2‐hydroxymethylenequinoline photocages described later are usually either 7‐hydroxyquinoline or 8‐aminoquinoline derivatives. In the case of these novel quinoline photocages the substrate scope has not been investigated in such detail as for BHQ or its 8‐cyano analogue. The benefits of the novel photocages were investigated in other regards, such as red‐shifted absorbance, increased photolysis efficiency or improved water solubility.

For fine‐tuning the photocleavage step it is crucial to have a good understanding of its mechanism. In the case of BHQ, a joint effort of experimental and computational methods led to initial proposals for the mechanism, which were later revised. The photocleavage mechanism of BHQ was initially studied by Stern‐Volmer quenching, time‐resolved IR (TRIR) and ^18^O‐labeling experiments. No statistically relevant changes in the uncaging were observed when measuring the 1PE quantum efficiency of BHQ‐OAc in the presence of different concentrations of triplet quenchers. In TRIR experiments (laser flash photolysis at 266 nm) stable products were formed also in the presence of oxygen, confirming the authors’ hypothesis that photolysis starts from a singlet excited state of BHQ‐OAc, while the triplet excited state of BHQ‐OAc decays back to the ground state without chemical transformation. Upon photolysis of BHQ‐OAc in H_2_^18^O, the BHQ‐OH product was exclusively labeled with ^18^O, indicating that the oxygen is added from the solvent (i.e., does not originate from the initial ester functionality via a simple hydrolytic pathway). Based on the experimental data, the authors proposed solvent‐assisted S_N_1 photoheterolysis as the reaction mechanism, similar to the process observed with Bhc or (7‐methoxycoumarin‐4‐yl)methyl (MCM) photocages [50]. The productive pathway, i.e. the O‐C bond photocleavage, proceeds from the singlet excited state by heterolytic or homolytic cleavage followed by single electron transfer, leading to a zwitterion‐like quinoline intermediate and the desired carboxylate substrate. The final trapping of the positive center by water yields the corresponding quinoline‐2‐methyl alcohol as a by‐product.

**Figure 6:**
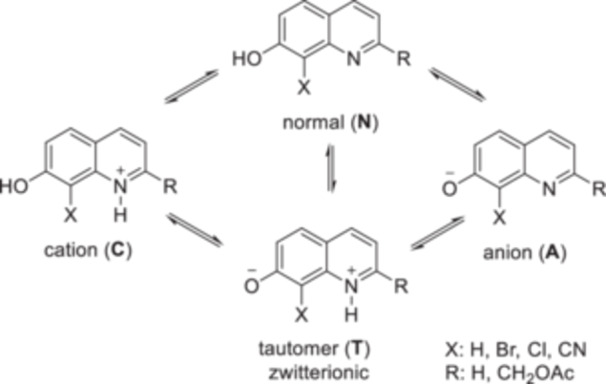
Equilibrium among the prototropic species of 7‐hydroxyquinoline or 8‐substituted‐7‐hydroxyquinolines in water.

To describe the photolysis mechanism, the ground state form should be considered first. 7‐Hydroyquinoline photocages contain both a proton donor and a proton acceptor site. 7‐Hydroxyquinoline itself is a well‐studied system whose spectroscopic properties in aqueous buffer solution have been investigated (e.g., [51]). In neutral aqueous solution, 7‐hydroxyquinoline is in an equilibrium of four prototropic species (one neutral, one anionic, one cationic and one zwitterionic/tautomeric form), which can interconvert by proton transfer (Figure 6). Upon photoirradiation, 7‐hydroxyquinoline undergoes excited state proton transfer (ESPT) via a water‐mediated proton‐relay system. In the photoexcited state, the p*K*_a_ of the 7‐OH group decreases, while that of the quinolinium nitrogen increases. Therefore, the 7‐OH group can act as a proton donor and the quinolinium nitrogen as a proton acceptor. This photoacidic character was later also rationalized by computational methods [52].

Ring substitution has a significant effect on the fluorescence of 7‐hydroxyquinoline, for example 4‐ethoxycarbonylmethyl‐7‐hydroxy‐2‐methylquinoline can be used as a green‐emitting (λ_max_ = 509 nm, Φ_f_ = 0.15) fluorescent dye. The substitution pattern also has a strong influence on the p*K*_a_ values and the prototropic equilibrium; the absorption and excitation spectra are strongly pH dependent. The substituent effect on the prototropic equilibrium of the ground‐state species of the 8‐substituted 7‐hydroxyquinolines BHQ‐OAc, 8‐cyano‐7‐hydroxyquinoline‐2‐ylmethyl acetyl ester (CyHQ‐OAc) and 8‐chloro‐7‐hydroxyquinoline‐2‐ylmethyl acetyl ester (CHQ‐OAc) was investigated using absorption and resonance Raman experiments [53, 54]. In addition, a thorough comparison with the parent 7‐hydroxyquinoline was also performed [55]. Like 7‐hydroxyquinoline, BHQ can exist in aqueous solutions in four prototropic forms: as a neutral, as an imine‐protonated cation, as an enol‐deprotonated anion and as an imine‐protonated plus enol‐deprotonated zwitterionic tautomer (Figure 6). The 8‐Br substituent in BHQ lowers the p*K*_a_ of the phenolic OH compared to 7‐hydroxyquinoline (from p*K*_a_ 9.0 to p*K*_a_ 6.8 in BHQ‐OAc) [47], therefore, different equilibrium proportions of the prototropic forms are expected [56]. To explain the substituent effects, the formation of cyclic hydroxyquinoline‐water complexes, hydrogen bonding between the substituent group and water, and the stereoelectronic effects of the substituents should also be considered. In the formation of the tautomeric form, i.e. in the proton transfer reactions, the solvent molecules play a role both in the ground state and in the excited state. The fluorescence spectra of BHQ‐OAc in neutral aqueous solutions were similar to those obtained in KMOPS buffer and were assigned to the singlet excited state of the tautomeric form. Since the excited anionic singlet species is the potential precursor of photodeprotection, it was therefore hypothesized that the ESPT process leading to the tautomeric form may be a competing process of photolysis of BHQ‐OAc in neutral aqueous solutions. Under certain conditions, a competing de‐bromination reaction could also take place.

In the case of CHQ‐OAc, which has the same p*K*_a_ as BHQ‐OAc (p*K*_a_ 6.8), the neutral species is the predominant form in neutral acetonitrile‐water mixed solution (pH 6–7). In the case of CyHQ‐OAc, which has a lower p*K*_a_ value (p*K*_a_ 4.9), both the neutral and anionic forms are present in the neutral aqueous mixed solution. The resonance Raman spectrum of CHQ‐OAc in neutral aqueous mixed solution indicates a larger proportion of the tautomeric form next to the neutral form than in the case of BHQ‐OAc, while the tautomeric form of CyHQ‐OAc is almost absent. According to density functional theory (DFT) calculations, the predominant transitions upon excitation lead to higher electron densities in the ring with the acetate group, which could favor its release.

**Figure 7:**
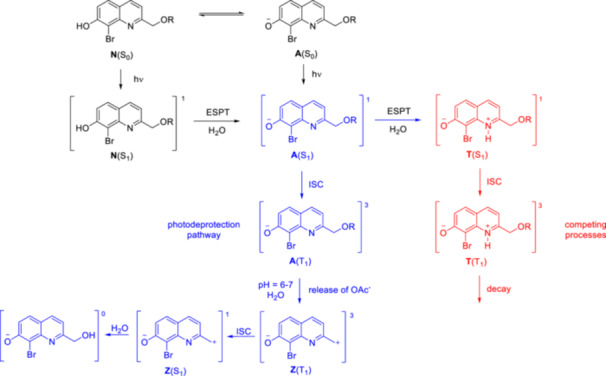
Photodeprotection mechanism of BHQ‐OAc in neutral aqueous solution [52, 57]. [Color figure can be viewed at wileyonlinelibrary.com]

The photodeprotection mechanism of BHQ‐OAc and CHQ‐OAc was further investigated under different solvent and pH conditions using transient emission (ns‐EM), transient absorption (ns‐TA), transient resonance Raman (ns‐TR^2^), and time‐resolved resonance Raman (ns‐TR^3^) spectroscopies [57]. As before, photolysis via a singlet transient species was initially proposed. Intuitively, CHQ‐OAc should have a higher 1PE quantum efficiency than BHQ‐OAc, as the bromine heavy atom could better support a competing intersystem crossing (ISC) process that degrades the singlet excited state. Experimentally, however, the opposite trend was observed. Repeated quenching experiments in neutral mixed aqueous solutions in the presence of excess potassium sorbate (PS) triplet quencher revealed an inhibition of the photochemical reaction. These results suggest that triplet species are involved as precursors in the photodeprotection, contrary to previous assumptions. In addition, a stronger emission was measured for CHQ‐OAc, i.e. fluorescence is a more important competing pathway. Based on laser excitation experiments, acetate release in neutral water‐containing solutions was found to occur on an ns timescale from an A(T_1_) precursor. Considering photodeprotection, ESPT leading to T(T_1_) is a competing process. An A(T_1_) precursor species for photodeprotection was also proposed for CHQ‐OAc. In addition, water and neutral pH were found to be important factors for a successful deprotection reaction. DFT calculations supported photolysis proceeding via heterolytic cleavage of A(T_1_) to form a triplet zwitterion‐like intermediate, subsequent ISC to the singlet state, and water solvolysis (Figure 7). Several reports have demonstrated that it may be a worthwhile strategy to develop PPGs that photorelease the payload by heterolytic bond dissociation in the triplet excited state [58]. Triplet state lifetimes are usually considerably longer than singlet excited state lifetimes, that allows surpassing energy barriers. PPG development supported by computational investigation of the excited states and the photorelease mechanism has been described for several PPG classes, such as BODIPY (boron‐dipyrromethene) [59] or heptamethine dye‐derived PPGs [60]. For quinoline PPGs, rational design based on computational studies was applied in several cases for 7‐hydroxyquinoline PPGs (*vide infra*).

To gain a deeper understanding of the direct photorelease of phenol substrates, the ESPT processes for BHQ‐protected phenol (BHQ‐OPh) in aqueous solutions were investigated using ultrafast (fs to ps) and fast (ns to ms) spectroscopic and DFT methods [61]. In agreement with previous observations, different ratios of prototropic (neutral and anionic) forms are obtained depending on pH and solvent. In acetonitrile/PBS (pH 7.4) 1:1, the anionic form is predominant, whereas upon photoirradiation the triplet (T_1_) anionic form was detected. The femtosecond transient absorption (fs‐TA) spectra in acetonitrile/PBS (pH 7.4) 1:1 showed the photoinduced excitation of the anionic form of BHQ‐OPh and the ISC from the anionic S_1_ to the T_1_ state. Based on these observations, photorelease from the T_1_ state was proposed for BHQ‐OPh at neutral pH, similar to BHQ‐OAc. Overall, the experimental and computational results suggest similar initial photoinduced and ESPT processes and photolysis mechanism in the case of BHQ‐protected phenols and BHQ‐protected acids. Investigation of the mechanism of photorelease of tertiary amines using the same photocage scaffolds led to controversial observations [62]. No significant change in Q_u_ values was observed in the presence of excess PS triplet quencher, and spectroscopic studies at 365 nm light irradiation also showed no reaction quenching. The discrepancy was attributed to the different wavelengths of light used in the different studies. Based on the results of the Stern‐Volmer quenching experiments, photorelease of CyHQ‐caged tertiary amines via a singlet excited state was proposed. If a triplet excited state is involved, it cannot be quenched by PS or sodium 2‐naphthalenesulfonate (SNS) due to its short lifetime.

### 2.1 Structure‐Property Relationships of BHQ and CyHQ

**Figure 8:**
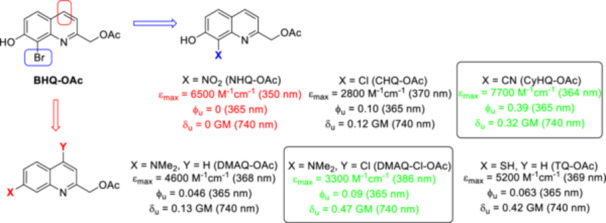
Structure‐property relationships of BHQ photocages. ε = 1P absorption, ϕ_u_ = 1P uncaging quantum yield, δ_u_ = 2P uncaging action cross section. [Color figure can be viewed at wileyonlinelibrary.com]

In a follow‐up study to the first report on BHQ, six novel chromophores were prepared to investigate how substituents on the quinoline ring affect the photophysical‐photochemical properties (Figure 8) [63]. Based on mechanistic assumptions, Br was replaced by other electron‐withdrawing (EWG) groups (Cl, CN, NO_2_), which can lower the p*K*_a_ of the phenol, facilitating the formation of the phenolate with greater *λ*_max_ and molar absorptivity, while not contributing to competing ISC processes. In addition, the thiol analogue of 7‐hydroxyquinoline was prepared. Due to the beneficial effect of changing OH to NMe_2_ in coumarin chromophores, an NMe_2_‐substitution was performed. The novel probes had similar or slightly shifted *λ*_max_ values as the BHQ‐OAc, but larger molar absorptivities. In aqueous KMOPS buffer, both bands corresponding to phenol and phenolate were detected in the UV‐Vis spectra of BHQ‐OAc and CHQ‐OAc, while only the phenolate band was observed in the case of 8‐nitro‐7‐hydroxyquinoline‐2‐ylmethyl acetyl ester (NHQ‐OAc) and CyHQ‐OAc. Compared to BHQ‐OAc, NHQ‐OAc and 7‐mercaptoquinoline‐2‐ylmethyl acetyl ester (TQ‐OAc) showed no fluorescence, while the other four chromophores showed stronger emissions compared to BHQ‐OAc. As for the 1PE sensitivities at 365 nm, CyHQ‐OAc outperformed the parent BHQ‐OAc, while the other chromophores showed lower activities. The two‐photon uncaging action cross sections (*δ*_u_) were lower than for BHQ‐OAc and did not directly correlate with 1 PE photocleavage. CyHQ‐OAc exhibited the highest 1 PE sensitivity, mainly due to its increased molar absorptivity, with its *δ*_u_ lower than that of BHQ‐OAc, while 7‐dimethylamino‐4‐chloroquinoline‐2‐ylmethyl acetyl ester (DMAQ‐Cl‐OAc) had the highest *δ*_u_ value and simultaneously low 1PE sensitivity. As a possible structural modification of 7‐hydroxyquinolines, the position of the electron‐donating (EDG) hydroxy group was changed from C7 to C6 to increase the stability of the assumed zwitterion‐like intermediate of photolysis. On the other hand, an aromatic (nitrogen) heterocycle was introduced with the aim of increasing the water solubility and improving the photochemical properties by extended conjugation. The (7‐hydroxy‐8‐(pyridin‐3‐yl)quinolin−2‐yl)methyl acetate (3’‐PyHQ‐OAc) probe exhibited low fluorescence and increased water solubility compared to BHQ‐OAc as well as increased photolysis kinetics; however, quantitative characterization of the novel probes has not been reported [64].

**Figure 9:**
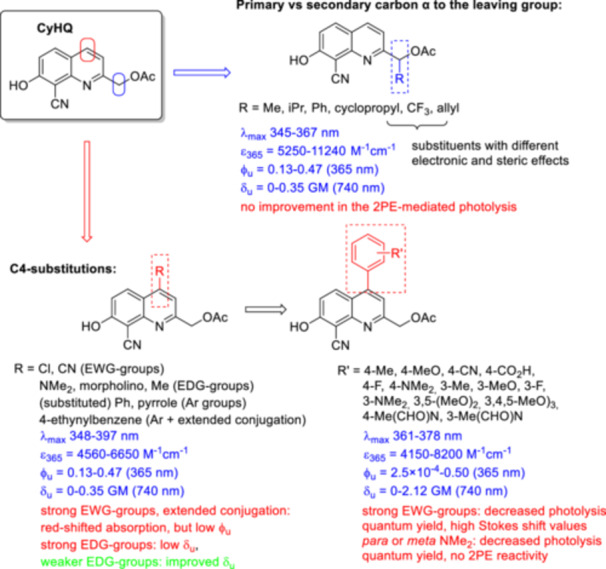
structure‐property relationships of CyHQ photocages. ε = 1P absorption, ϕ_u_ = 1P uncaging quantum yield, δ_u_ = 2P uncaging action cross section. [Color figure can be viewed at wileyonlinelibrary.com]

The effect of different chemical modifications on the photochemical properties, in particular the δ_u_ values was investigated in detail for the CyHQ photocage [65]. Of the two parameters (δ_a_ and Φ_u_) that affect δ_u_, it is easier to fine‐tune the two‐photon absorption term (δ_a_) by specific structural modifications, such as extending conjugation, introducing molecular symmetry, constructing multibranched oligomers, introducing planarity or positioning strong donor/acceptor pairs [10, 66]. Computational methods could also facilitate the optimization of absorption spectra. However, modifications aimed at increasing 2P absorption (2PA) generally resulted in bulkier, lipophilic structures with suboptimal water solubility. Improving two‐photon photolysis is more elusive, as the fate of the excited state is difficult to predict and depends on several factors (*vide supra*—Figure 2), such as the structure of the substrate or the photolysis conditions, in particular the solvent, pH or ionic content. Therefore, the development of novel 2PE PPGs is often based on trial‐and‐error cycles [67]. However, the Φ_u_ photolysis term can be strongly influenced even by minor changes. One of the modifications tested was the introduction of a substituent at the 2‐methyl position, turning the primary carbon next to the leaving group into a secondary carbon (Figure 9). The photochemical properties were investigated in 0.1 mM solutions of the probes in physiological buffer (pH 7.2 KMOPS) with 365 nm (1PE) LED light or 740 nm (2PE) Ti:sapphire laser light. Of the six examples prepared, an improvement in δ_u_ was observed in one case (with isopropyl group), but overall this direction was not considered promising. As another alternative, modifications in the C4 position, i.e. in the *meta* position versus the leaving group, were performed to utilize the selective transmission of the electron density of an aromatic ring in the first excited state (“Zimmerman *meta*‐effect”) [68, 69, 70, 71], which could accelerate the rate of light‐induced heterolysis. To cover various electronic properties, EWG, EDG and aromatic groups were investigated. The effect of an aryl ring with different electronic properties in the C4 position was further explored by introducing differently substituted aryl groups. A 4‐ethynylbenzene derivative was also prepared to test the effect of extended conjugation. Of the 22 new derivatives investigated, the C4 4‐MePh and the C4 3,5‐(MeO)_2_‐Ph were the two best performers in terms of photolysis efficiency and sensitivity to 2PE. However, the C4 3,4,5‐(MeO)_3_‐Ph analogue [(8‐cyano‐7‐hydroxy‐4‐(3,4,5‐trimethoxyphenyl)‐quinolin‐2‐yl)methyl acetate—TMP‐CyHQ‐OAc] was also characterized by high hydrolytic stability in the dark as well as high quantum yield and 2PE sensitivity. Plotting the sensitivity (ε × Φ_u_) of the novel PPGs against the Hammet constants (σ) of the aryl substituents reveals a good correlation (*R* ^2^ = 0.81), i.e. a positive effect originating from an electron‐rich phenyl group. Consistent with the previously proposed photolysis mechanism, i.e. the formation of a positive charge on C2‐methylene upon photolysis, the transmission of electron density from the *meta* position could contribute to the stabilization of the cation. A correlation (*R* ^2^ = 0.45) was also suggested between δ_u_ and the Hammet constants. Importantly, good chemical yields (63%–92%) were obtained with the best performing probes for the release of the UV‐active homopiperonylate payload.

### 2.2 Structure‐Property Relationships of 8‐DMAQ

**Figure 10:**
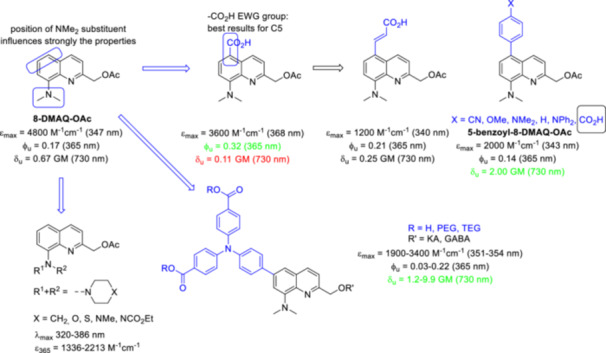
Structure‐property relationships of 8‐DMAQ photocages. ε = 1P absorption, ϕ_u_ = 1P uncaging quantum yield, δ_u_ = 2P uncaging action cross section. [Color figure can be viewed at wileyonlinelibrary.com]

Inspired by previous results showing a clear influence of the substitution pattern on the photophysical and photochemical properties of quinoline PPGs, a systematic study on the correlation between photofragmentation and the position of the NMe_2_ group was performed [67]. Interestingly, no photofragmentation was observed for 5‐ and 6‐dimethylaminoquinoline‐2‐ylmethyl acetyl ester (5‐ and 6‐DMAQ‐OAc) at 365 nm within 2 h of photoirradiation. However, an increased rate of photofragmentation was measured for 8‐dimethylaminoquinoline‐2‐ylmethyl acetyl ester (8‐DMAQ‐OAc) compared to 7‐dimethylaminoquinoline‐2‐ylmethyl acetyl ester (7‐DMAQ‐OAc). Similar trends were observed for 2PE photofragmentation at 730 nm. In two‐photon excited fluorescence (TPEF) experiments, 5‐, 6‐, and 7‐DMAQ‐OAc showed modest maximum 2PA cross section values in the NIR region, in contrast to 8‐DMAQ‐OAc, which exhibited the highest 730 nm 2PA cross section of the four regioisomers. The C8 NMe_2_ can be replaced by other cyclic amino groups without negatively affecting the photochemical properties. This could pave the way for the development of, e.g., bifunctional photoactivatable building blocks [72]. Subsequently, further systematic modifications of the 8‐DMAQ PPG were investigated (Figure 10). Firstly, the effect of an additional EWG group on photolysis was investigated, based on the assumption that strong donor−acceptor pairs contribute to increased 2PA via internal charge transfer (ICT) [10, 73]. The EWG of choice, a carboxylate, was also expected to lead to better water solubility. Based on the 1PE photolysis results, the C5 position clearly appeared to be the most suitable for the introduction of an EWG‐substituent. Interestingly, the enhanced 1PE photolysis of the 5‐carboxy‐8‐DMAQ‐OAc analogue did not translate into enhanced 2PE photolysis. The C5‐dipole was further elaborated by extending the π‐conjugation via an olefin or an aryl moiety. While the extended π‐conjugation did not prove fruitful for 1PE photolysis, the 5‐benzoyl‐8‐DMAQ‐OAc far outperformed the parent 8‐DMAQ‐OAc under 2PE conditions. Inspired by the promising results obtained with the 5‐carboxyphenyl substitution, the effect of an aryl ring with various electronic properties in position C5 was explored by introducing differently substituted groups [74]. The photochemical properties were studied in 0.1 mM solutions in acetonitrile/TRIS buffer 1:1 (pH 7.4), with 365 nm (1PE) UV‐light or 730 nm (2PE) Ti:sapphire laser light. Four of the novel probes showed increased sensitivity and 1PE photolysis efficiency compared to 5‐benzoyl‐8‐DMAQ‐OAc, but their 2PE sensitivity was less remarkable. Still, two of the probes had decent δ_u_ values (0.63–0.64 GM). In the case of the 5‐cyanophenyl‐8‐DMAQ‐OAc the increased 2PE performance compared to the parent 5‐phenyl‐8‐DMAQ‐OAc was attributed to the push‐pull system within an extended π‐conjugated system, while in the case of 5‐dimethylaminophenyl‐8‐DMAQ‐OAc, synergistic effect between the EDG group and the extended conjugated system was suggested. In both cases, an overall enhancement of ICT was assumed. Intracellular photorelease of a payload via photochemical internalization was targeted with an 8‐DMAQ PPG. For this aim, the 8‐DMAQ PPG was functionalized with a triphenylamine photosensitizer decorated with further solubilizing groups (dicarboxylic acids, di‐PEG esters and di‐TEG esters) in C6 position [75]. As an additional benefit, the triphenylamine EDG can induce intramolecular donor to acceptor charge transfer upon photoexcitation in D‐A type molecules and thereby lead to enhanced absorption. Interestingly, positioning the triphenylamine in position C5 did not result in improved photochemical properties [74]. The novel PPG was tested for the controlled release of kainate and GABA, that were attached via ester or carbamate linker. The absorption data showed no significant electronic coupling or conjugation between the triphenylamine and the DMAQ scaffold, while the molar extinction increased by one order of magnitude. The calculated 2PE uncaging values for in vitro GABA or kainate release were much higher than that of the parent 8‐DMAQ‐OAc (in the range of 1.2–9.9 GM). Intracellular release was investigated by using a rhodamine 110 reporter substrate. The fluorescence of the reporter is quenched by binding the PPG and it can be restored upon photodeprotection. HeLa cells were incubated with the reporter‐PPG construct and free extracellular rhodamine liberated by intracellular esterases was removed by replacing the medium before photoirradiation. Following 357 ± 44 nm light irradiation (DAPI [4′,6‐diamidino‐2‐phenylindole] dye excitation filter), efficient fluorescent staining of the cells was observed, that confirmed the cellular uptake and the intracellular photocleavage of the probe. Of note, efficient photorelease could also be operated under 2PE conditions at 760 nm.

### 2.3 *N*‐Methylquinolinium PPGs

**Figure 11:**
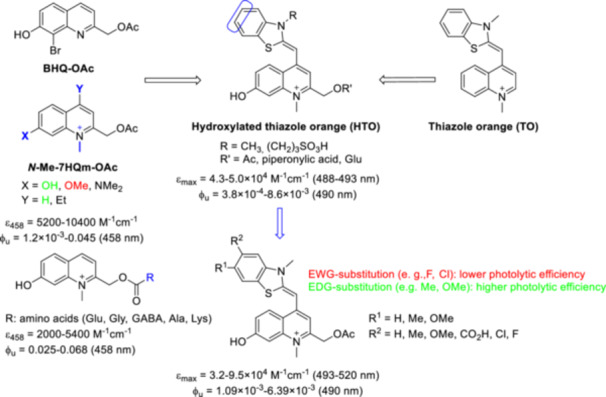
Structure‐property relationships of *N*‐methylquinolinium photocages. ε = 1P absorption, ϕ_u_ = 1P uncaging quantum yield, δ_u_ = 2P uncaging action cross section. [Color figure can be viewed at wileyonlinelibrary.com]

Based on the photophysical data of *N*‐alkyl‐7‐hydroxyquinolinium derivatives [76], it was predicted that *N*‐alkylation of quinoline PPGs leads to a red‐shifted absorption maximum (> 400 nm, i.e., already in the Vis region), an increased extinction coefficient and a higher water solubility without negatively influencing the photolysis quantum efficiency [77]. Furthermore, this structural change could enable late‐stage functionalization after conjugation of the substrate. These design considerations have also been successfully implemented, for example, in red‐shifted, π‐extended coumarin PPGs [78] or fluorescent dyes [79]. Although other alkyl groups also modulated favorably the photophysical properties among quinolines, *N*‐methylation was studied in more detail (Figure 11). When comparing a small set of 7‐substituted chromophores, the *N*‐methyl‐7‐hydroxymethylquinolinium (*N*‐Me‐7‐HQm) probe proved to be the best performer with increased 1PE photolysis sensitivity (studied in PBS buffer at pH 7.2 with 458 nm LED‐light). Due to the hydrolytic instability of the ester bond, the novel probes were used to prepare a series of caged amino acids via a carbamate linker with the substrate. The *N*‐methylation step was performed after conjugation of the appropriately protected amino acids (Glu, Gly, GABA, Ala, and Lys), i.e. as the last step of the synthetic sequence, followed by a final deprotection step. Both the parent *N*‐Me‐7‐HQm‐OAc and the *N*‐Me‐7‐HQm‐caged amino acids had high water solubility (~10–20 mM in PBS/water). Since the authors' main goal was to produce Vis‐activatable probes, the 2PE properties of the novel cages were not investigated. Inspired by the *N*‐Me‐7‐HQm chromophore, the green‐light absorbing thiazole orange (TO) fluorescent dye was converted into a 2‐hydroxymethylenequinoline PPG scaffold (Sul‐HTO). In addition to merging the PPG and fluorescent dye scaffolds, additional hydrophilic groups were introduced to improve water solubility [80]. Photolysis was studied using acetic acid and UV‐active piperonylic acid substrates with green light irradiation. Interestingly, the released acetic acid was also monitored with ^1^H NMR. The 1PE uncaging cross section exceeded that of the PPG construct with BODIPY core analogue, which was attributed to higher Φ_u_ values. The novel chromophore was used to prepare a long wavelength‐light sensitive caged glutamate (Sul‐HTO‐Glu). The Sul‐HTO‐Glu probe could be efficiently photolyzed under both 1PE and 2PE conditions (at 490 or 940 nm, respectively), i.e. at longer wavelengths not covered by previously described quinoline PPGs. However, 2PE photolysis was characterized by the percentage release of the substrates, with no specific δ_u_ values reported. Based on spectroscopic and ^18^O‐labeling studies, the authors suspected a similar photodeprotection mechanism as in the case of BHQ‐OAc. For a control experiment, the PPG was prepared without the 7‐OH group. Its reduced photolysis showed the essential role of the 7‐OH group for the photochemical efficiency. The properties of the HTO scaffold were further systematically adjusted by introducing different EWG and EDG substituents via late‐stage functionalization on the benzothiazole ring and investigating their effects on the photolytic process [81]. The structural changes were expected to have an effect on ICT upon photoinduced excitation. The trends observed in the spectroscopic properties were explained by the effect of the substituents on the frontier orbitals. Since the electron density of the highest occupied molecular orbital (HOMO) is concentrated on the benzothiazole ring, its substitution by EWG‐groups leads to larger HOMO‐LUMO (lowest unoccupied molecular orbital) gaps via the stabilization of the HOMO orbital (i.e., a higher absorption energy) [82]. Therefore, a blue shift would be expected in the light absorption, which is consistent with experimental observation. Conversely, EDG‐groups contribute to a red‐shifted absorption. EDG‐substituted HTOs had a higher photolytic efficiency than the unsubstituted HTO, while EWG substitution decreased the photolytic efficiency. When the photolytic efficiency is plotted against the transferred charge (obtained from DFT calculations) of the HTO derivatives, a good correlation (*R* ^2^ = 0.87) is obtained, confirming the logic of the design principles of the novel probes.

### 2.4 Quinoline PPGs With Fused Ring Systems

**Figure 12:**
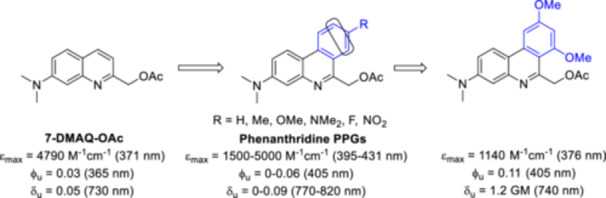
Structure‐property relationships of phenanthridine‐based photocages. ε = 1P absorption, ϕ_u_ = 1P uncaging quantum yield, δ_u_ = 2P uncaging action cross section. [Color figure can be viewed at wileyonlinelibrary.com]

Structural changes usually affect the substitution pattern of the quinoline ring, not the ring itself. In a recent work, a “benzannulation” approach was tested: a small series of 3‐(dimethylamino)‐phenanthridines (3‐DMAPh) was prepared (Figure 12) [83]. The novel chromophores exhibited absorption maxima around 400 nm, i.e. a value clearly red‐shifted compared to the parent 7‐DMAQ‐OAc, but had lower emission coefficients. Based on calculations (DFT and time‐dependent DFT [TD‐DFT]), a correlation between EDG groups and higher Φ_u_ values was found. In TPEF experiments, the EWG/EDG‐substituents did not significantly alter the 2PA cross sections, which remained in the range of the parent DMAQ chromophores. However, the substituents led to a bathochromic shift of the 2PA maximum. The 2PE photolysis results remained modest, so further optimization was performed aided by DFT‐calculations (screening charge transfer distance (D_CT_) values) to determine the optimal D–π–A–π–D’ substitution pattern. The most promising probe showed a two‐photon sensitivity of 1.2 GM, which is a 20‐fold increase over the 3‐DMAPh derivative without additional groups.

### 2.5 Quinoline Probes With Increased Structural Complexity: Octupolar and Quadrupolar Probes

**Figure 13:**
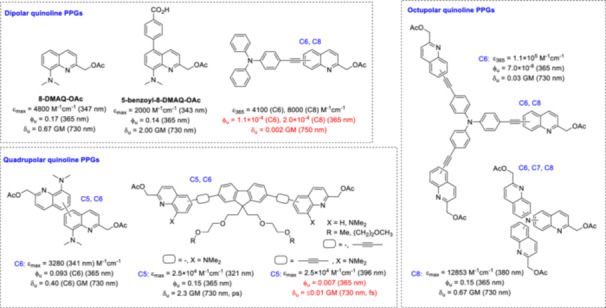
Dipolar, quadrupolar, and octupolar quinoline derivatives. ε = 1P absorption, ϕ_u_ = 1P uncaging quantum yield, δ_u_ = 2P uncaging action cross section. [Color figure can be viewed at wileyonlinelibrary.com]

Several attempts have been made to integrate quinoline cages into multipolar (i.e., dipolar, octupolar or quadrupolar) systems and to investigate the 2PE photolysis sensitivity of these constructs. A more comprehensive discussion of 2PE selection rules and molecular symmetry is beyond the scope of this review; we refer readers to reviews that address specifically these aspects (e.g., [10, 26, 84, 85, 86, 87, 88]. A structure‐property study on 2PE chromophores verified experimentally the hypothesis that symmetric charge transfer upon excitation from the ends to the center of a conjugated system or vice versa is correlated with 2PA enhancement [89]. A general dipolar 2PA chromophore consists of two electron donor or acceptor groups connected to a central core scaffold via conjugated systems. In the case of a quadrupolar chromophore geometry, the electron donor or acceptor groups are directly bound to the central core scaffold. The 2PA properties can be modulated either by extended conjugation or by increasing the EDG or EWG effect of the respective end groups. In a PPG context, a non‐centrosymmetric PPG was developed with a planar central fluorenyl core (a chromophore with high 2PA cross section) [90] substituted with PEG chains to compensate for lipophilicity and functionalized with two nitrobenzyl PPG units linked via C–C double bonds [(2,7‐bis‐(4‐nitro‐8‐[3‐(2‐propyl)‐styryl])‐9,9‐bis‐[1‐(3,6‐dioxaheptyl)]‐fluorene, BNSF] [91, 92]. The BNSF PPG releases glutamate efficiently in the 740–850 nm range, with a 2PE uncaging action cross‐section of 2 GM. This design was adapted for a quadrupolar quinoline chromophore (Figure 13) [93]. The novel chromophore showed a modest 2PA in the 700–900 nm range and two orders of magnitude lower uncaging quantum yield than the corresponding dipolar derivative with the same PPG unit in 1PE photolysis experiments. The quadrupolar donor‐neutral‐donor (D‐N‐D) triad probe design was applied with 8‐DMAQ PPG units connected to the central pegylated fluorenyl core via the C5 or C6 position [94]. Despite lower molar extinction coefficients, the probes with the DMAQ units directly connected to the central fluorene showed faster 1PE photolysis. However, no 2PE photolysis was detected at a pulse width of 150 fs at 730 nm, i.e. the experimental results did not correlate with the theoretical δ_u_ values, suggesting different photochemical pathways under 1PE and 2PE conditions. When the pulse width parameter (1.05 ps pulses) was changed, the novel probes exhibited δ_u_ values in the range of 1.3–2.3 GM. The effects of excited state absorption (ESA) and intermediate state resonance enhancement (ISRE) were proposed to explain this experimental observation. To evaluate C_2_‐symmetric chromophores quadrupolar probes were prepared by directly grafting two 8‐DMAQ units via their C5 or C6 position [95]. The smaller differences observed in the UV‐Vis absorbance of the novel probes compared to 8‐DMAQ‐OAc indicated low electronic coupling between the branches. The directly grafted dimers showed decreased photolysis compared to 8‐DMAQ‐OAc under both 1PE and 2PE conditions, with the C5 regioisomer performing slightly better (in agreement with the results on 5‐aryl‐8‐DMAQ probes) [74]. Since in centrosymmetric chromophores a reversal in selection rules between 1PA and 2PA is expected (i.e., the first excited electronic transition is 2PE‐forbidden) [66], the surprisingly decent 2PE photolysis of the dimers could be explained by conformational flexibility and symmetry breaking.

Dipolar and donor‐centered tripodal octupolar derivatives were designed combining a triphenylamine EDG moiety with EWG quinoline PPG moieties via an ethynyl spacer [93]. Due to coupling between the dipolar branches, a similar design (i.e., octupolar chromophores built around a triphenylamine) led to 2PA at higher energy (2PE‐allowed, but 1PE‐forbidden electronic state) in previous cases. The new probes had high molar extinction coefficients (greater than the corresponding DMAQ analogues) and significant fluorescence. Increasing the polarity of the solvent resulted in a positive solvatochromic behavior: a bathochromic shift in the emission spectra, increasing the Stokes‐shift, consistent with an ICT transition with large dipole moment increase upon excitation. Quantitative analysis of the solvatochromic behavior of dipolar and octupolar compounds supported a strong increase of the dipole moment in the excited state. Dipolar derivatives showed increased 2PA compared to the monomeric analogues, and an even more pronounced 2PA enhancement was observed for the octupolar chromophores, with the C6 regioisomers performing better than the C8 regioisomers in this respect. In 1PE photolysis, modest uncaging quantum yields were observed for the dipolar derivatives and even lower performance for the octupolar probes. Conversely, an opposite trend was hypothesized for the 2PE uncaging quantum yield, with the octupolar probes exhibiting δ_u_ values an order of magnitude higher than the dipolar probes due to their higher 2PA. However, the calculated δ_u_ values were still in the range of 0.03–0.04 GM. Of note, related 8‐Br‐quinoline derivatives with a (diphenylamino)phenyl‐ethynyl group in the C6 position were studied as 2P photosensitizers and exhibited significant 2PA cross‐sections, efficient singlet oxygen production and strong fluorescence [96]. Structurally, the C6 extension and the bromine heavy atom at the C8 position facilitate ISC and singlet oxygen generation. Due to their photophysical‐photochemical properties, the novel chromophores could be used as sensitizers for dual photodynamic therapy (PDT)/fluorescence imaging. Tripodal structures built around a donor nitrogen (i.e., no conjugated bridges) were investigated, thereby introducing a third‐order rotational symmetry [97]. 2PA enhancement was expected from a cooperative synergistic effect between the branches through electronic interactions. In the case of the C6 and C7 regioisomers, competitive carbazole formation was detected under 365 nm photoirradiation. Sequential photorelease of the model acetic acid was observed for the C8 isomer. However, despite an increased 2PA, the 2PE uncaging action cross section of the C8‐tripode did not exceed that of the monomeric dipolar 8‐DMAQ. It should be noted that additional challenges may arise in molecular engineering aimed at nanostructuring around dendrimeric architectures, such as the occurrence of competing photochemical pathways or the occurence of π‐stacking interactions between the branches that reduce the efficiency of the monomeric units [98].

## 3 Applications

For characterizing novel quinoline PPGs, typically acetic acid is used as the model substrate. In the following section, the controlled release of biologically relevant substrates using quinoline PPGs is exemplified. In several cases, the applicability of the novel constructs was verified also in cellular or in vivo experiments. Of note, photoactivation under biologically more relevant 2PE conditions was efficiently used for various objectives.

### 3.1 Release of Biologically Relevant Phenols

**Figure 14:**
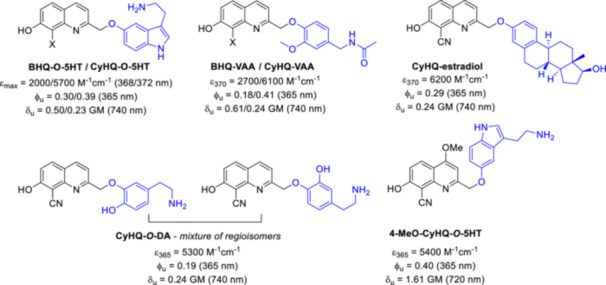
Examples for biologically relevant phenols caged by quinoline PPGs. ε = 1P absorption, ϕ_u_ = 1P uncaging quantum yield, δ_u_ = 2P uncaging action cross section. [Color figure can be viewed at wileyonlinelibrary.com]

Phenol functionalities are ubiquitous, but not a typical site for the introduction of PPGs. Several protecting groups require carbonate binding, and in photocleavage the additional decarboxylation step negatively affects the release rate of the substrate. Direct binding of the PPG to the phenolic OH is a more favorable option. When a BHQ construct with phenol model substrate was investigated, it photolyzed with a quantum efficiency of 0.19 at 365 nm and a 2PE photolysis cross section of 0.56 GM at 740 nm in KMOPS buffer, demonstrating that phenols can be viable substrates for the BHQ platform [99]. Of the wide range of biologically active phenols, serotonin (5‐hydroxytryptamine, 5‐HT) is of particular interest due to its various biological roles, e.g. as a neurotransmitter or hormone. For preparing photoactivatable serotonin, both the NH_2_ and the phenolic OH function were used in previous constructs for the attachment of the PPG. For a comparative study, both caging options were also tested with BHQ (BHQ‐*O*‐5HT with a phenol ether and BHQ‐*N*‐5HT with a carbamate linker) [99]. BHQ‐*O*‐5HT and BHQ‐*N*‐5HT each efficiently released serotonin under 1PE (370 nm) or 2PE (740 nm) conditions, even when compared to known PPG‐serotonin constructs. The biological relevance of BHQ‐*O*‐5HT photorelease was investigated by extracellular recordings from dissociated sensory neurons prepared from mouse dorsal root ganglia and trigeminal ganglion or optic tectum in intact zebrafish larvae. The neuronal response was first recorded with 5‐HT before testing that BHQ‐*O*‐5HT itself or the PPG without the payload are not active. After a light pulse of 365 nm, activity comparable to 5‐HT was recorded with BHQ‐*O*‐5HT. Importantly, a cell viability assay showed that mammalian neurons tolerate BHQ‐*O*‐5HT well. In in vivo experiments with zebrafish larvae, photolysis of BHQ‐*O*‐5HT using 365 nm light resulted in similar changes in electrographic activity as the parent compound 5‐HT, and repeated light exposure resulted in repeated induction of neural activity. The physiological effect of BHQ‐*O*‐5HT was investigated in the study of left‐right (LR) patterning in *Xenopus laevis* embryos [99, 100]. After photolysis, LR defects including situs inversus and heterotaxia were observed in BHQ‐*O*‐5HT‐injected embryos to a similar extent as in 5‐HT treatment. Importantly, low toxicity was observed also in this system following BHQ‐*O*‐5HT treatment. The direct linkage of phenol substrates to PPG has been extended to other substrates and quinoline PPGs. In this context, not only BHQ but also CyHQ was tested (Figure 14) [101]. In addition to serotonin, the insect neurotransmitter octopamine, capsaicin, *N*‐vanillylnonanoylamide (VNA), estradiol, and tyrosine were selected as biologically relevant phenol substrates. Consistent with previous results, the CyHQ‐protected phenols exhibited a higher molar absorption compared to the BHQ‐protected series. Under simulated physiological conditions at 365 nm, the CyHQ‐series exhibited an average quantum efficiency of 0.36, which is slightly higher than the average value of 0.29 for the BHQ‐series. Interestingly, an opposite trend was observed for 2PE photolysis at 740 nm: the BHQ‐series exhibited average δ_u_ values of 0.55 GM, while the CyHQ‐series exhibited 0.25 GM. Moreover, in the case of BHQ‐protected estradiol, a non‐productive but not unprecedented debromination side reaction was observed instead of substrate photorelease. Debromination is influenced by the leaving group character of the payload, so substrates with a higher p*K*_a_ (such as estradiol) are more prone to this type of side reaction. CyHQ‐protected dopamine and the D_2_ and D_3_ selective dopamine receptor antagonist sulpiride were investigated in the context of studying dopamine signaling and the kinetics of receptor activation and inactivation [102]. Dopamine has three functionalities (two phenolic OH and a primary NH_2_ group) that could serve as potential sites for PPG linkage. To investigate the efficacy of the different protection strategies, four dopamine (DA)‐PPG constructs were prepared that block one or two of the functional groups: CyHQ‐*O*‐DA, (CyHQ)_2_‐*O*,*O*‐DA, CyHQ‐*N*‐DA, and (CyHQ)_2_‐*N*,*O*‐DA (in the case of CyHQ‐*O*‐DA and (CyHQ)_2_‐*N*,*O*‐DA a mixture of the regioisomeric ethers was isolated). In the case of sulpiride, the tertiary amine moiety was used to introduce the PPG (*vide infra*). The novel constructs exhibited strong absorption in the 365 nm range and had a tail that extended above 405 nm so that they could also be activated with standard lasers at 405 nm. The novel constructs had 0.19–0.20 1PE Φ_u_ values at 365/405 nm, with the exception of (CyHQ)_2_‐*N*,*O*‐DA. Three of the constructs photolyzed under 2PE conditions, with 0.12–0.26 δ_u_ values. Despite the decent results for 1PE quantum yield and 2PE uncaging action action cross‐section, (CyHQ)_2_‐*O*,*O*‐DA showed a modest chemical yield for the released DA, likely due to the two independent photolysis reactions required. A modest chemical and quantum yield was also observed for (CyHQ)_2_‐*N*,*O*‐DA. The photochemical properties of CyHQ‐sulpiride were similar to those of other CyHQ‐protected tertiary amines. The novel constructs exhibited sufficient hydrolytic solubility and stability for biological testing. CyHQ‐*O*‐DA was selected for biological studies due to its clean and efficient photorelease reaction with fast release kinetics. CyHQ‐*O*‐DA was first tested in a cell culture of MDA‐MB‐231 breast cancer cells expressing D_1_ receptors. Activation of the receptors leads to an intracellular [Ca^2+^] increase, which can be detected with the fluorescence indicator Fluo‐4 AM. After irradiation with 405 nm light, the fluorescence intensity was measured, confirming a similar response for CyHQ‐*O*‐DA + light as in the case of free DA. Midbrain slice preparations from the substantia nigra pars compacta express D_2_ receptors that activate the G‐protein‐coupled inwardly rectifying potassium (GIRK) channel. To monitor receptor activation, K^+^ currents can be measured with voltage clamp recordings on whole cell. Flash photolysis of CyHQ‐*O*‐DA resulted in a GIRK‐mediated response that could be blocked with the D_2_ receptor antagonist sulpiride. The kinetics of the CyHQ‐*O*‐DA photolysis response indicate an effective DA concentration in the micromolar range. Repeated photoactivation could also be performed with CyHQ‐*O*‐DA. CyHQ‐sulpiride was tested to measure the decay constants of receptor signaling after photorelease of the antagonist in brain slices expressing D_2_ receptors. It showed no significant background activity (a challenge for antagonists with nanomolar activity) and was efficient in assessing the off rates of DA or quinpirole agonists.

In subsequent work, the CyHQ core was substituted in position C4 to improve its 2PE cross section [103]. For the first studies DA was used as model phenol substrate. The molar absorptions at 365 nm were in similar range as that observed for the parent CyHQ and all studied probes released DA with moderate to good yields under 1PE conditions. In contrast with previous results with acetate substrate, the PPGs with an aromatic C4 substituent showed slower photolysis than CyHQ‐*O*‐DA. This interesting experimental observation in the case of phenol substrates was explained by a PET process between the catechol ring of DA and the aromatic C4 substituent, that competes with the photochemical reaction in the excited state. On the other hand, significant rate enhancement was observed with MeO or morpholino substituents in the C4 position. Interestingly, no 2PE photolysis was detected for TMP‐CyHQ‐*O*‐DA, although it was an efficient PPG for acetic acid. 4‐MeO‐CyHQ‐*O*‐DA was identified as the best performing 2PE PPG for DA, with a sixfold higher δ_u_ than that of CyHQ‐*O*‐DA. However, 4‐MeO‐CyHQ‐*O*‐DA exhibited a blue‐shifted λ_max_. The most efficient 4‐MeO‐CyHQ PPG was tested for the release of a series of biologically relevant phenols: DA, 5‐HT, the synthetic DA agonist rotigotine, as well as VNA and eugenol, two natural products exhibiting transient receptor potential (TRP) ion channel activator activity. 4‐MeO‐CyHQ released efficiently the phenol substrates both under 1PE and 2PE conditions, except for rotigotine. In this latter case, it was suggested that the thiophene ring in the substrate may trap potential energy in its excited state that quenches afterwards the photochemical reaction. The release of DA from 4‐MeO‐CyHQ‐*O*‐DA was evaluated in HCT116 cells expressing the dLight 1.2 fluorescent sensor. Upon exposure to 405 nm (1PE) or 720 nm (2PE) light pulses, a correlation was observed between the fluorescent response, the delivered light pulse, and the presence of the PPG construct. Similarly, 1PE‐ and 2PE‐induced 5‐HT release from 4‐MeO‐CyHQ‐*O*‐5HT was demonstrated in HCT116 cells expressing the GRAB_5‐HT_ sensor. The 1PE and 2PE release of VNA and eugenol was studied in HEK293 cells expressing TRPV1 receptors, with Fluo‐4 calcium‐sensitive dye monitoring Ca^2+^ influx. As eugenol and VNA are odorants, their caged version can be used for chemosensory research. In this context, the caging of other odorants was tested with CyHQ PPG [104].

### 3.2 Release of Biologically Relevant Tertiary Amines

**Figure 15:**
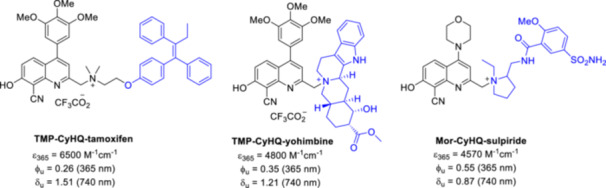
Examples for biologically relevant phenols caged by quinoline PPGs. ε = 1P absorption, ϕ_u_ = 1P uncaging quantum yield, δ_u_ = 2P uncaging action cross section. [Color figure can be viewed at wileyonlinelibrary.com]

The scope of CyHQ PPG was extended to the photorelease of biologically relevant tertiary amines (Figure 15), another less common functionality for the binding of PPGs [62]. The p*K*_a_ of the leaving group was previously considered crucial for the photorelease. However, the p*K*_a_ of ammonium and alkylammonium ions should theoretically allow their release from a quaternary ammonium salt of CyHQ. The CyHQ‐protected quaternary amines bearing a permanent positive charge showed good solubility (> 0.1 mM) in KMOPS buffer, except for the constructs prepared with the more lipophilic tamoxifen and 4‐hydroxytamoxifen payloads. The novel constructs exhibited a λ_max_ around 370 nm, indicating that the probes are predominantly in the phenolate form at physiological pH. Depending on the structure of the amine, moderate to good 1PE (365 nm) quantum efficiencies were measured. However, no photorelease was observed for aromatic amines (i.e. amines with sp^2^ hybridization) despite the low p*K*_a_ values of the conjugate acids.

**Figure 16:**
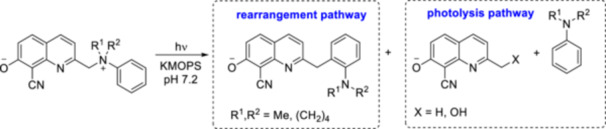
Photorearrangement reaction of quinoline‐protected dialkylanilines. [Color figure can be viewed at wileyonlinelibrary.com]

Interestingly, in the case of CyHQ‐protected anilines, exposure to light resulted in an intramolecular photoaza‐Claisen rearrangement, later classified as Hofmann−Martius‐type rearrangement, instead of photorelease of the amines. This unexpected rearrangement was optimized into a synthetic reaction, which produces substituted anilines. It could proceed via the formation of the cationic intermediate after cleavage of the C−N bond in the excited‐state. During the rearrangement, the cation is trapped by the aryl ring instead of water. To better understand the mechanism of the photorearrangement, the properties of the ground‐state were studied with UV‐Vis absorption and Raman spectroscopy and *in silico*, while the properties of the excited state after light irradiation were investigated with fs and ns time‐resolved TA spectroscopies [105]. The results obtained with the CyHQ construct of *N,N*‐dimethyl‐2‐phenylethan‐1‐amine indicate the generation of the S_1_ excited state, C–N bond cleavage and ISC. The photolysis product originates from the reaction between the longer lifetime triplet excited state of CyHQ^+^ and water (of note, the photorelease mechanism proposed is often revised as novel data are emerging, *vide supra*). Further investigation of the scope, mechanism, and structure−activity relationship (through modifications of the aniline ring, *N,N’*‐dialkyl groups, and CyHQ) identified the structural factors by which the rearrangement versus photorelease process can be shifted towards the photorelease of dialkylanilines (Figure 16) [106]. The photolysis pathway generated the corresponding quinaldine and benzyl alcohol derivatives of CyHQ. It is noteworthy that the formation of quinaldine has not been observed previously during CyHQ‐photocleavage in aqueous media. In the modifications on the aniline ring, bulky substituents in the *meta* or *para* position increased the yield of photolysis products. In the modifications of CyHQ at the 2‐methyl position, the introduction of a methyl group led to a clear shift in the process towards photolysis.

Photorelease from modified CyHQ PPG was demonstrated with the progesterone receptor antagonist steroid mifepristone (RU‐486). The formation of substantial amount of the *N*‐demethylated mifepristone analogue metapristone (RU‐42633) indicates the presence of radical species in the excited state and an alternative α‐elimination pathway after C−N bond cleavage. The 1PE‐activatable PPG‐tertiary amine constructs released tertiary amines also under 2PE conditions, with an average cross‐section of 0.30 GM, i.e. in the same range as CyHQ‐protected acetate or phenols. TD‐DFT, UV and resonance Raman spectra showed that CyHQ‐caged triethylamine (CyHQ‐TEA) is mainly in the oxyanionic form under neutral aqueous conditions and mainly in the neutral form in acetonitrile. The different ground state precursors depending on the solvent can lead to different photochemical and photophysical processes after photoexcitation. Using ultrafast broadband time resolved fluorescence (TRF) and TA, the singlet excited states, the conversion between different electronic states and the lifetime constants (τ) for the non‐radiative steps were investigated. The estimated time constant for the cleavage of the C‐N bond from a singlet state intermediate is 70 ps, which is significantly faster than the acetate release from BHQ‐OAc. Structural modifications of CyHQ, which have been shown to be fruitful in the context of phenol substrate photorelease, were investigated for the release of tertiary amines [107]. Namely, substitutions on C4 with EDG groups with different electronic properties were tested. The four constructs investigated released the sulpiride model substrate with high 1PE sensitivity and good chemical yields. The C4‐substituted probes exhibited higher δ_u_ compared to CyHQ‐sulpiride, with the most significant (eightfold) improvement observed in the case of TMP‐CyHQ‐sulpiride. The potential of TMP‐CyHQ as a PPG for tertiary amines was demonstrated for a number of simple, complex and biologically relevant amines, such as the anticancer drug and gene expression regulator tamoxifen and its active metabolite 4‐hydroxytamoxifen or the natural alkaloid yohimbine.

### 3.3 Optical Control of Oligonucleotide Activity and Gene Expression With Quinoline PPGs

**Figure 17:**
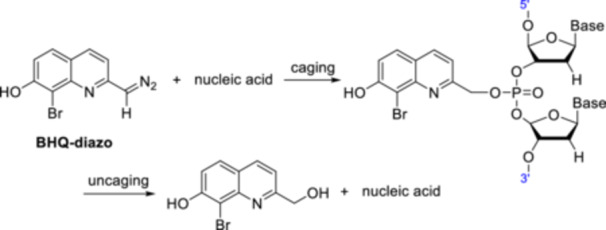
Photoregulation of aptamer activity with quinoline PPGs. [Color figure can be viewed at wileyonlinelibrary.com]

(Bromo)‐hydroxyquinoline PPGs were used to photocontrol aptamer activity (Figure 17) [108]. The thrombin‐ and prothrombin‐binding HD1, a 15mer ssDNA aptamer, was chosen as the target substrate [109]. To introduce the protecting group, HD1 was co‐incubated with the diazomethane form of the PPGs (i.e., a non‐site‐specific, multiple caging strategy was used). To select the most suitable PPG (i.e., the one with the highest photolysis sensitivity as well as efficient covalent bond formation with the phosphate moiety of the sugar‐phosphate backbone of the nucleic acid), a series of PPGs with Br and OH substituents in different positions was investigated. Based on the experimental results, BHQ was the most promising PPG for this application, which released the caged HD1 with good conversion when irradiated with 365 nm light. The effect of caging on the specific binding of HD1 to its target was investigated by surface plasmon resonance. Introducing BHQ PPG decreased the binding to the target by 88%, which could be restored to more than 85% compared to the parent aptamer after photoirradiation. Using the same co‐incubation protocol for binding the PPG to nucleic acids, the diazomethane form of BHQ was used to control the plasmid expression of green fluorescent protein (GFP) as a model protein [110]. After demonstrating caging and photolysis in vitro, the expression level of BHQ‐caged GFP was examined in vivo in HeLa cells transfected with the caged and native plasmids.

**Figure 18:**
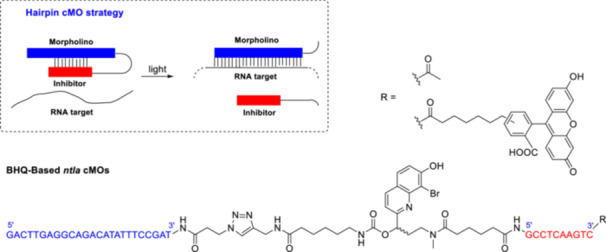
Photoactivatable hairpin morpholino oligonucleotides with quinoline PPGs. [Color figure can be viewed at wileyonlinelibrary.com]

Synthetic morpholino oligonucleotides (MOs) are valuable experimental antisense tools for temporary gene silencing in zebrafish. MOs have a neutral backbone of non‐natural morpholino rings linked by phosphorodiamidate bonds [111, 112]. MOs are resistant to nucleases; however, their constitutive activity limits their use. Photoactivatable MOs could be used to precisely control the timing and location of gene silencing, allowing tissue‐ and stage‐selective study of embryonic development. In a potential caged MO (cMO) design, a complementary MO‐derived inhibitor was attached to the target sequence via a photocleavable linker in a hairpin structure (Figure 18) [113]. Photocleavage leads to dissociation of the inhibitor, allowing the MO to interact with the target RNA. After detailed characterization and structure‐activity studies of hairpin cMOs with a DMNB PPG, cMOs with a BHQ PPG were studied to investigate 2PE photocleavage of the antisense reagents. Conditional silencing of the T‐box transcription factor *no tail‐a* (*ntla*) was chosen as a model system, since disruption of *ntla* function in zebrafish leads to distinct morphological phenotypes. A BHQ derivative with an *N*‐hydroxysuccinimide ester and a terminal alkyne side chain was prepared as a bifunctional reagent. The combination of cMO treatment with light irradiation in wild‐type zebrafish embryos resulted in the *ntla* phenotype, demonstrating the applicability of the novel probe. Importantly, BHQ‐based *ntla* cMO was capable of inducing *ntla*‐related morphological changes in target regions of zebrafish embryos at 820 nm 2PE. On the downside, the efficacy of the hairpin design depends on the linker structure as well as the sequence and position of the complementary inhibitor.

**Figure 19:**
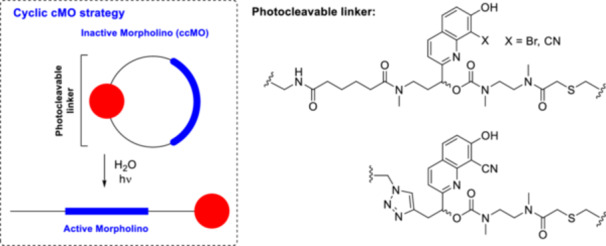
Photoactivatable cyclic morpholino oligonucleotides with quinoline PPGs. [Color figure can be viewed at wileyonlinelibrary.com]

As another oligonucleotide caging strategy, a cyclic cMO (ccMO) was designed, in which the PPG is positioned in the linker through which the MO is cyclized (i.e., between the two 5′ and 3′ ends of the sequence) [114]. Cyclization reduces the RNA hybridization capacity of the ccMO to the mRNA target. Therefore, a PPG with two arms is also required in this case. ccMOs with BHQ and CyHQ PPGs were prepared against the glutamic acid decarboxylase *gad1b* and *gad2* genes to bind to the translation start sites (Figure 19) [115]. Glutamic acid decarboxylases are involved in the synthesis of GABA from glutamic acid and therefore play an essential role in GABA‐ergic signaling and in its multiple biological functions. Photolysis at 365 or 405 nm linearized the MOs by cleavage of the linker and facilitated binding to the complementary mRNA. The ccMOs showed relevant stability in the dark for prolonged experiments in the presence of buffers or degrading enzymes. The applicability of the new ccMOs for studying the role of specific genes in later stages of zebrafish development was demonstrated in in vivo experiments with BHQ‐*gad1b*‐ccMO and CyHQ‐*gad1b*‐ccMO. While early injection of *gad1b*‐MOs caused severe craniofacial morphological defects, zebrafish treated with ccMOs and reared in the dark developed normally. Abnormal electrophysiological brain activity was observed when the ccMOs were later light‐activated compared to wild type animals. In a later work, a novel, synthetically more accessible linker design based on the Huisgen 1,3‐dipolar cycloaddition was proposed for the coupling reaction with the oligonucleotide, with an ethynyl functionality in the PPG side chain [116]. In addition to the higher yield of the synthetic sequence, the triazole linkage in this case also had the advantage of improved stability in the dark. The novel ccMOs are efficiently photolyzed under UV illumination (405 nm, LED), and in in vitro DNA hybridization experiments, photoactivation led to a 5.8‐fold increase in baseline activity.

**Figure 20:**
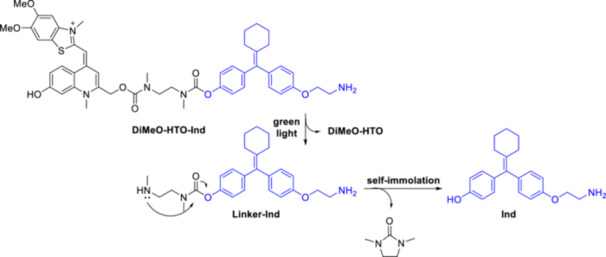
DiMeO‐HTO caged cyclofen‐OH inducer for green light‐triggered DNA recombination. [Color figure can be viewed at wileyonlinelibrary.com]

Dimethoxy‐substituted HTO PPG was used for optical control of DNA recombination by caging cyclofen‐OH, a stable alternative to caged tamoxifen‐OH, which is susceptible to various photochemical side reactions [117]. The substrate was attached via a self‐immolating dimethylethylenediamine‐carbamoyl linker (resulting in the caged **ind**ucer DiMeO‐HTO‐**Ind** construct) (Figure 20) [81]. The free cyclofen‐OH inducer is efficiently released with 490 nm green light. Induction of the gene recombination by DiMeO‐HTO‐Ind in living cells was confirmed in HEK293T cells transfected with Cre‐ERT2 and loxP‐mCherry‐loxP‐BFP plasmids (i.e., by using a fluorescent reporter). The Cre‐ERT2/loxP system is widely used for studying DNA recombination activated by small molecule inducers. Cre‐ERT2 encodes a Cre recombinase fused to an estrogen ligand‐binding domain (ERT2) that is activated in the presence of tamoxifen. In the model system, after binding of the ER‐ligand (cyclofen‐OH) to Cre‐ERT2, the loxP‐flanked mCherry is deleted by Cre, leading to expression of the blue fluorescent protein (BFP). Photorelease of the effector after irradiation with green light was assessed by monitoring BFP expression using fluorescence microscopy and flow cytometry. Treatment with DiMeO‐HTO‐Ind in combination with green light resulted in increased BFP fluorescence and expression.

### 3.4 Investigation of Neurological Processes

**Figure 21:**
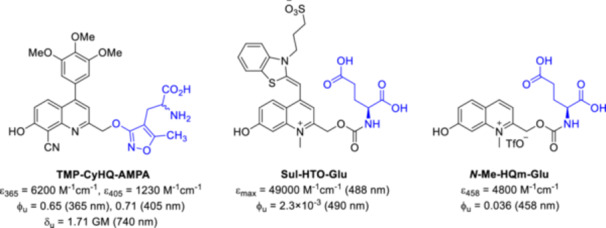
Neuroactive amino acids caged by quinoline PPGs. ε = 1P absorption, ϕ_u_ = 1P uncaging quantum yield, δ_u_ = 2P uncaging action cross section. [Color figure can be viewed at wileyonlinelibrary.com]

Due to the controlled, rapid jump in effector concentration that can be driven with PPGs, the agonists and antagonists of neuronal receptors are prime substrates for PPGs [20]. PPGs have been used primarily as experimental tools for the controlled release of neurotransmitters or neuroactive amino acids. Several PPGs have been tested for glutamate in particular [118, 119], and some of these constructs are also commercially available. The application of caged glutamates is limited by several challenges, such as the low 2P uncaging action cross section (δ_u_), slow release kinetics that do not match the biological process studied, suboptimal hydrolytic stability and poor solubility in water. At higher concentrations, caged glutamates can act as antagonists of GABA‐ergic transmission [120]. To circumvent the latter problem, AMPA (α‐amino‐3‐hydroxy‐5‐methyl‐4‐isoxazolepropionic acid) can serve as a surrogate for the study of excitatory transmission [121]. Ionotropic AMPA receptors are one of the glutamate receptor subtypes where AMPA is a selective agonist. A CyHQ‐based PPG was selected for the development of a 2PE‐activatable AMPA construct (Figure 21) [122]. In a previous caging approach, the PPG was bound to the NH_2_ group of AMPA via a carbamate linkage [121]. However, with carbamates, photorelease is a two‐step process, with rapid photolysis of the C–O bond and slow decarboxylation of the carbamic acid intermediate, leading to photodeprotection in the millisecond to second timescale. To achieve faster photorelease, in the novel design the PPG was attached to the 3‐hydroxy group of the isoxazole ring, as CyHQ PPGs have been shown to efficiently release phenols [101]. TMP‐CyHQ‐AMPA exhibited excellent (> 100 μM) solubility in KMOPS buffer and efficiently released AMPA under 1PE (365 or 405 nm) and 2PE (740 nm) conditions, with an increased 1.71 GM δ_u_ value. To investigate whether the PPG efficiently blocked the affinity of AMPA to the receptor, molecular docking simulations were performed. However, no further pharmacological validation was carried out. Another highly potent agonist that can be used instead of glutamate to avoid an antagonistic effect on the GABA‐A receptor is kainic acid. Kainate is a partial agonist with high affinity at kainate‐type glutamate receptors and lower affinity at AMPA‐type glutamate receptors. A detailed comparison of MNI‐Glu and MNI–kainate (MNI–KA) revealed lower current responses for MNI‐KA, yet caged kainates may be useful tools for functional mapping of kainate receptor localization [123]. For KA caging a 5‐benzoyl‐8‐DMAQ PPG was used [74]. Of note, unlike with glutamate, no hydrolytic stability issues were recorded. Short bursts of photolysis elicited large inward currents and somatic spikes in Purkinje neurons.

Glutamate itself was caged with 8‐DMAQ [67]. Photorelease was tested on neuronal preparations with 1 ms flashlamp (300–380 nm) photolysis in cerebellar slices from 20‐day postnatal rats using whole‐cell patch‐clamp recordings. Using a 1 mM concentration of 8‐DMAQ‐Glu, photorelease of glutamate resulted in rapidly increasing responses with similar amplitude and rise‐time as glutamate released by MNI‐Glu under the same conditions. However, the ester linkage between the PPG and glutamate was not sufficiently hydrolitically stable. Glutamate was photocaged with a HTO PPG as well [80]—with the aim of developing a long wavelength‐light‐sensitive caged glutamate. In Sul‐HTO‐Glu, an additional sulfonic acid was introduced to increase water solubility, and caging was accomplished via carbamate binding to the Glu‐NH_2_. Remarkably, Sul‐HTO‐Glu could be efficiently photolyzed under 1PE conditions with green light at 490 nm (with a maximum yield of 40%). The 2PE photolysis of Sul‐HTO‐Glu was evaluated by comparing with the 2PE photolysis of commercially available MNI‐Glu at 740 nm. A 1‐h irradiation resulted in a significantly higher amount of free glutamate in the case of the novel probe (37% vs. 7% from MNI‐Glu). To evaluate the applicability in a biological setting, the activation of glutamate‐gated ion channels (NMDAR) was investigated in *Xenopus* oocytes and HEK293T cells. In the former system, ion currents resulting from glutamate binding were recorded using voltage clamp. Short light irradiation (505 nm LED) elicited an inward current in NMDAR‐expressing *Xenopus* oocytes, which was not observed in the absence of Sul‐HTO‐Glu. In HEK293T cells, Ca^2+^ influx was monitored via NMDARs with a red fluorescent Ca^2+^ indicator. Short light irradiation (505 nm LED) triggered Ca^2+^ influx in Sul‐HTO‐Glu treated cells, while the PPG construct or its photoproducts showed no cytotoxicity. The *N*‐Me‐HQm PPG was tested to cage a range of amino acids: Glu, Gly, GABA, Ala, and Lys [77]. Upon binding the PPG to the α‐carboxyl group of glutamic acid, a stability problem emerged, presumably due to low hydrolytic stability. Therefore, caging via a carbamate linkage at the NH_2_ group was tested. The 1PE photolytic efficiency of *N*‐Me‐HQm‐caged Glu did not exceed that of commercially available RuBi‐Glu, but was still high enough for practical applications.

### 3.5 Quinoline‐Functionalized Photoactivatable Nanoparticles

**Figure 22:**
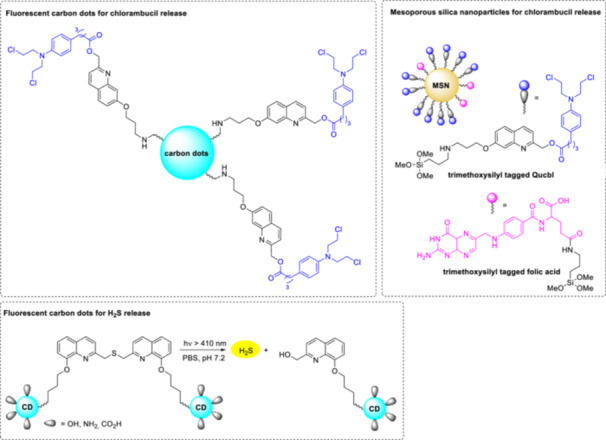
Photoactivatable nanoparticles for targeted substrate release with a quinoline PPG unit. [Color figure can be viewed at wileyonlinelibrary.com]

Quinoline PPGs have been incorporated as photoactivatable components in photoresponsive nano drug delivery systems (DDS). DDSs consisting of biocompatible fluorescent carbon dots (Cdots) and 7‐hydroxyquinoline PPG units covalently anchored to the surface of the nanoparticles were designed to enable simultaneous fluorescent imaging and regulated delivery of anticancer drugs (Figure 22) [124]. Chlorambucil (cbl) bound to the PPG (Qucbl) via an ester bond was chosen as model anticancer drug. Under 1PE (365 nm) and 2PE (632 nm) conditions, 73% and 20% drug release from Qucbl‐Cdots was measured, corresponding to 0.29 and 0.17 photochemical quantum yields for the parent Qucbl and the functionalized Qucbl‐Cdots, respectively. The cellular uptake of Qucbl‐Cdots and their nuclear localization in HeLa cells was confirmed by fluorescence monitoring and nuclear staining experiments. When the in vitro cytotoxicity of Qucbl‐Cdots was evaluated using the MTT [3‐(4,5‐dimethylthiazol−2‐yl)‐2,5‐diphenyltetrazolium bromide] assay, an increase in cytotoxicity was observed at a light exposure of 365 nm, which was confirmed by cell cycle analysis. In a control experiment, no significant cytotoxicity was observed at light exposure in the presence of unfunctionalized Cdots. In another work, cellular and nuclear targeted charge reversal photoresponsive nanoparticles were designed using the same 7‐hydroxyquinoline PPG‐chlorambucil conjugate [125]. Protonation of the quinoline ring under slightly acidic conditions was used for charge reversal. Biocompatible mesoporous silica nanoparticles (MSN) decorated with folic acid were selected as nanocarriers, as they can be easily taken up by cells and have a high drug loading capacity. The Qucbl conjugate was covalently grafted onto the surface of the MSNs using a silane coupling agent. Studies on cellular localization of Qucbl‐Fol‐MSNs by time‐dependent confocal laser scanning microscopy (CLSM) confirmed a pH‐dependent uptake. Furthermore, efficient folic acid targeting was observed, i.e. increased cellular uptake was detected in cancerous HeLa cells overexpressing folic acid receptors compared to normal cells. The Qucbl‐Fol‐MSNs localized dominantly in acidic organelles. The anticancer drug was efficiently released under 1PE (365 nm) or 2PE (675 nm) conditions, consistent with a previous report [124]. In vitro cytotoxicity was measured in HeLa cells using the MTT assay. The Qucbl‐Fol‐MSNs and the MSN nanocarrier were relatively nontoxic but decreased cell viability was observed after 365 nm (23% cell viability) or 675 nm (73% cell viability) light irradiation. In another design, 8‐hydroxyquinoline PPG units covalently linked to fluorescent carbon dots were used for targeted photorelease of the gaseous signaling molecule H_2_S [126]. QuH_2_S‐Cdots showed a broad absorption band (300–500 nm) and a maximum emission at 425 nm. H_2_S photorelease from QuH_2_S‐Cdots in PBS buffer (pH ~7.4) at 410 nm was quantified using an H_2_S−sensitive *N,N*‐dimethylaniline‐hemicyanine fluorescent probe. Quantification of H_2_S release was also performed using the methylene blue assay. Cellular H_2_S release with 410 nm light from QuH_2_S‐Cdots was measured in HeLa cells by confocal microscopy. The biocompatibility of the nanocarrier system was confirmed by evaluating the cytotoxicity in HeLa cells using the MTT assay.

In the series of nanostructures, quinoline PPGs were tested in a microfluidic context by integrating the PPGs into surfactants composed of PEG/perfluorinated polyether (PFPE) diblock amphiphiles [127]. An 8‐piperazino derivative was selected as the photocleavable aminoquinoline linker because it could be functionalized at two sites (C2 hydroxymethyl and C8 amino moieties). Both possible designs were tested (i.e., the PFPE chain attached to the piperazine and the PEG chain to the 2‐hydroxymethyl or vice versa), but only one of them proved to be experimentally feasible. Using 355 nm picosecond pulsed laser light, targeted microdroplets could be selectively and rapidly merged in the range of seconds.

**Figure 23:**
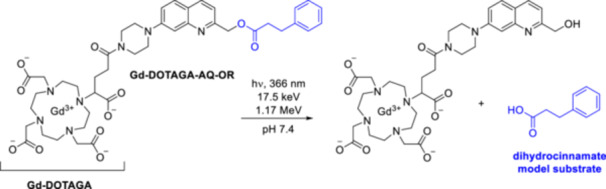
X‐ray ligand release from a quinoline PPG‐based system. [Color figure can be viewed at wileyonlinelibrary.com]

Targeting less easily accessible body regions is a major bottleneck for DDSs activated by an external stimulus [128]. An interesting delivery system based on the photoactivation of organic probes by X‐rays has been described that should enable the controlled release of a covalently bound payload in deeper tissues (Figure 23) [129]. The underlying hypothesis was that in the presence of Auger electrons generated by the interaction of heavy metals with X‐ray or γ‐rays, a radical anion similar to that formed under photoexcitation can be generated, subsequently leading to products analogous to those formed during 1PE or 2PE photolysis. In the model system, a gadolinium (III) complex (the known MRI contrast agent, Gd(III)‐DOTAGA [1,4,7,10‐tetraazacyclo‐dodecane‐1‐glutaric acid‐4,7,10‐triacetic acid]) was used as the intramolecular heavy metal antenna and a 7‐aminoquinoline as the PPG unit. The photolysis and radiolysis of the model system were investigated under UV, X‐ray (17.5 keV) and γ irradiation (1.17 MeV) conditions. Under 1PE, the Gd antenna reduced the sensitivity of the PPG to photolysis but conferred high water solubility to the system. In a control experiment, the 7‐aminoquinoline PPG itself was not sensitive to radiolysis, while the full construct yielded the expected photolysis/radiolysis products upon irradiation. However, despite the promising proof of concept results, the efficiency of the Gd‐DOTAGA‐AQ system is not yet sufficient for long‐term applications in vivo, as the irradiation doses required to release the payload are beyond the lethal dosage range.

## 4 Current Challenges of Quinoline PPGs

In the 20 years since their first discovery, 2‐hydroxymethylquinoline PPGs have been thoroughly investigated in many ways. In addition to synthetic efforts to explore their structure‐property relationships, the photorelease mechanism of 8‐substituted‐7‐hydroxyquinolines has been extensively studied both experimentally and by computational methods. In this regard, less effort has been made to investigate the photorelease process of 8‐dimethylaminoquinolines. Quinoline PPGs can be efficiently used for Vis‐range or 2PE photolysis. Especially in recent years, several novel chemical tools with increased 2PE sensitivity have been described. Due to the large amount of experimental data, quinoline PPGs can serve as an interesting case study to investigate how the originally described PPG has evolved in different directions. Another important advantage of the quinoline platform is that it can be used for caging various functional groups, including less obvious ones (e.g., phenolic OH, tertiary amine). The availability of sites suitable for the formation of bonds with the PPGs is an important factor in the design of PPG‐mediated photoactivation experiments. This chemical consideration also contributed to a counter‐selection of effector molecules chosen for PPG‐based studies [106]. The applicability of the novel quinoline PPGs has been demonstrated in a wider range of cellular and in vivo studies. However, these reports are mostly from the groups working on the chemical development of the probes. This situation is not uncommon in the field of PPG development. A significant proportion of the biological/pharmacological studies using PPGs are based on a limited number of commercially available probes. The reason for the slow uptake of novel probes, even those with significantly improved profiles, can be explained by several factors. In the following section we would like to point out some potential obstacles.

i. **Standardization of experimental data, comparison of PPGs**
  As far as photorelease properties are concerned, PPGs are usually characterized by two numerical values: the 1PE uncaging quantum yield (Q_u_) and the 2PE uncaging action cross section (δ_u_). One would think that a simple comparison of these values would be sufficient to draw conclusions about PPGs. However, direct comparisons are often problematic. The 1PE uncaging quantum yield is given by the molar extinction coefficient, the time in which 90% of the reaction is completed, and the intensity of the light source used [Φ_u_ = (I × σ × t_90%_)^−1^]. A review of the literature shows that there is a wide variety of protocols and light sources used, so standardization of the experimental results is not easy. Lamp intensity, expressed in Einstein cm^−2^ s^−1^, can be measured by chemical actinometry. Chemical actinometers are chemical systems in which a light‐induced reaction takes place for which the quantum yield is known [130]. The quantum yield of a photochemical reaction is the number of events divided by the number of absorbed photons of a given wavelength in the same time period. Measuring the relevant reaction rate allows the absorbed photon flux to be calculated. In chemical actinometers, the photochemical conversion is directly related to the number of photons absorbed. Well‐established chemical actinometers can provide accurate and reproducible light intensity data when used correctly. However, due to the various analytical steps of an actinometer protocol, these can lead to measurement errors. An established chemical actinometer should fulfill strict criteria; in the field of PPGs, ferrioxalate actinometry (Hatchard‐Parker actinometer) is typically used [131]. It can be used in the wavelength range of 250–500 nm. The method is based on the irreversible light‐induced redox reaction of ferrioxalate, i.e. the light‐induced conversion of Fe^3+^ into Fe^2+^ and the measurement of the absorbance of the Fe^2+^‐1,10‐phenanthroline complex in buffered acidic solution at 510 nm. Due to the available analytical measurement step (spectrophotometry) and the availability of quantum yield values, ferrioxalate actinometry is widely used and has also been adapted, for example, to determine photon flux in photochemical flow reactors [132, 133]. In a standard laboratory environment, the following potential sources of measurement error may occur: (i) the experiment should be performed under dim red light (i.e., excluding ambient light), using freshly prepared and mixed solutions, (ii) the complexation step should be performed immediately after light irradiation by adding the irradiated solutions to a pre‐mixed buffer‐phenanthroline solution, (iii) sufficient time should be allowed for complexation, (iv) the phenanthroline solution itself is also UV‐sensitive, (v) high conversion to Fe^2+^ may exceed the complexation capacity of phenanthroline, (vi) linearity of results with irradiation time should be ensured, (vii) as several experimental conditions have been proposed in the literature, the protocol used should be clearly stated when publishing experimental results. Given the list of potential measurement errors and the often laconic reports of light intensity measurements in the experimental sections or supporting information of journal articles, reproducibility and comparison of results from different groups can sometimes be challenging. When comparing photolysis results, it is also important to consider the reaction medium used. Although experiments in aqueous medium are best suited for biological studies, the solubility of PPGs often necessitates the use of an organic co‐solvent. However, the ratio of water to organic solvent has a significant impact on the uncaging efficiency [81, 93].
  Regarding the efficiency of 2PE photolysis, the most reliable data can be obtained by direct measurement of photolysis under 2PE conditions; the 2P uncaging action cross section (δ_u_) corresponds to the product of 2PA (δ_a_) and uncaging quantum yield (Φ_u2_) (δ_u_ = δ_a_ × Φ_u2_). Depending on the access to suitable laser setups, quantification is often based on calculated δ_u_ values instead. Despite the analogy to 1PE processes, there are some important differences in 2PE processes. Firstly, 2PE processes are strongly dependent on the excitation power density. Therefore, the excitation geometry should be known exactly. Secondly, the often low 2PA of probes and the resulting suboptimal signal‐to‐noise ratio is another potential source of measurement error. The 2PA cross section can be measured directly using the z‐scan method by recording the absorption of the sample as the focal zone of a femtosecond laser is moved through the optical axis. Another possible method is TPEF imaging. TPEF utilizes the fluorescence signal induced by 2PA and provides the TPEF action cross section by comparison with either a reference compound or the 1PE fluorescence of the sample compound [85]. With a known δ_a_ value, the 2P uncaging can be estimated by assuming that ϕ_u2P_ = ϕ_u1P_. The latter can be conveniently determined under 1PE irradiation conditions, typically with HPLC or LC‐MS monitoring of the decay of the PPG construct. PPGs have many overlapping vibrational levels due to their more complex structure, allowing internal conversion to the lowest excited state. In the case of 2PE, if the photochemistry proceeds from the same excited state as in 1PE (Kasha rule), the estimation based on 1PE Φ_u_ may be a relevant option. However, we also found exceptions to this rule even in the present review. Finally, indirect measurements can also be made if the effector elicits a response that can be closely monitored and measured (e.g., an electrophysiological response triggered by a neurotransmitter) [134]. As mentioned above, 2PE uncaging is also influenced by several factors, making a direct comparison of literature results difficult. Apart from the numerical characterization data, the most important question is whether the chemical tool is suitable for a meaningful investigation of the given research question. This depends on a variety of properties that have been analysed by several authors (e.g. solubility, stability, biological inertness, photorelease kinetics) [6]. There are no universally applicable tools, and different thresholds may apply depending on the specific experiment.
ii. **Synthetic accessibility of PPGs**

**Figure 24:**
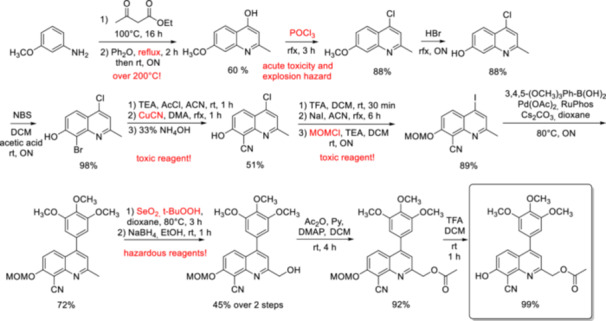
Synthesis of TMP‐CyHQ‐OAc [65]. [Color figure can be viewed at wileyonlinelibrary.com]

For the design of a PPG‐based experiment, the synthetic accessibility of the probe cannot be overemphasized. Commercial reagents are overrepresented because of their proven applicability, but also because of their availability. Biology/pharmacology research groups often do not have access to synthetic expertize and facilities. Synthetic accessibility is probably one of the shortcomings of the quinoline platform. On the one hand, the small molecule quinoline scaffold can be modified or functionalized in many ways using established chemical procedures (e.g., ring synthesis, cross‐coupling or S_N_Ar reactions), and as we have seen from the examples above, even small changes can lead to dramatic effects on photochemical behavior. However, the synthesis of the various PPGs is often a multistep process with a modest overall yield that involves technically challenging or hazardous steps (as illustrated by the preparation of TMP‐CyHQ‐OAc on Figure 24). In this regard, chemists play a crucial role by pursuing robust, greener and operationally simple synthesis alternatives. Quinoline PPGs are not (yet) commercially widely available (we have found a BHQ‐*O*‐5HT catalogue item though, at a biotech company). In this regard, a thorough needs analysis could guide which substrate‐PPG constructs are actively used and required by potential end users.

iii. **On‐demand development of novel chemical tools**
  Although there is a general consensus on the common challenges of PPGs that need to be addressed (e.g., activation in the biological window, improved 2PE properties, broad substrate range, clean and well‐established photorelease reaction), novel PPGs with an improved profile are slowly being taken up by other research groups. One question that needs to be asked is whether there is a real demand from end users for the novel probes developed by chemists. One potential challenge is that chemical development is often”disconnected” from end users. Novel PPGs are designed and characterized seemingly per se without targeting specific applications. A reverse process, i.e. a specific demand from users, could lead to case‐specific, intelligent chemical tools. However, on‐demand development of PPG constructs necessitates the close collaboration of experts of different disciplines.
iv. **Translational aspects of novel PPGs**

PPGs were originally developed as experimental tools. Recently there has been a growing number of prodrug applications, where PPGs are linked to drug molecules and applied for various in vitro and in vivo interventions. Since PPGs can increase the benefit/risk ratio of pharmacotherapy, it has long been discussed whether PPGs could enter 1‐day human therapy [8, 135]. Of the various challenges that a DDS which is activatable with an external light stimulus is facing [136], here we discuss only two aspects: light delivery and biosafety.

For in vivo light activation the “phototherapeutic window” (i.e., the 650–900 nm wavelength range) should ideally be targeted. Of the different alternatives, probably the easiest is to use PPGs that absorb and photorelease their payload in this window. This in itself is already a considerable challenge [137]. For quinoline PPGs, usually *N*‐alkylation has been used to red‐shift the absorption of the probes. The 2P activation property of several quinoline photocages is more relevant for experimental settings at present. For future clinical translation, several engineering aspects will also need to be considered, namely how the light is delivered to the targeted tissues for applications in deeper lying regions. As a feasible option for this issue, considerable effort is dedicated to the development of miniaturized wearable or implantable light‐emitting devices. Importantly, several case studies have reported the use of these devices for various in vivo photopharmacological interventions in animal models [138, 139].

In the case of PPG‐drug constructs, further levels of complexity are added to the already complex drug development process. A recent perspective gave a detailed overview of the challenges that need to be addressed for the further translation of photopharmacology using azo arene photoswitches [140]. Several of the challenges identified in the perspective are also relevant for PPGs, such as the “drug‐like” properties of the photoactivatable probe (e.g., polarity, planarity, penetration), balance of ADME‐pharmacokinetic properties or toxicology. In the case of 1:1 drug‐PPG constructs, after the photorelease step the products formed from the PPG will be present in stochiometric amounts. During the characterization of novel PPGs, usually cytotoxicity is studied in cellular assays, or the acute toxicity is tested for in vivo animal models. The metabolism of drug molecules with a quinoline scaffold have been investigated in detail. In addition, several reports describe the metabolism of quinoline itself or the metabolism of various quinoline derivatives [141, 142]. However, the metabolic transformation and toxicity of quinoline PPGs in different models has not yet been studied in detail. Such data is scarce also for other important PPG chemical subtypes. Further preclinical data on the metabolic and toxicological liabilities of different PPGs are needed to assess the translation potential of PPG‐drug constructs.

## 5 Concluding Remarks and Future Perspectives

The 2‐hydroxymethylquinoline platform was first described 20 years ago as a useful 2PE PPG. Over the past two decades, it has been credibly demonstrated that quinoline PPGs can be valuable tools for studying a variety of biological processes, from neuronal signaling to targeted delivery of anticancer drugs. Quinoline PPGs has the capability to be used for investigating niche applications, such as targeting specific subcellular organelles [143]. Given the complex structure‐property relationships uncovered, there is still considerable scope for further chemical developments, such as the merged scaffold of the HTO photocage, inspired by a fluorescent probe. Due to the large number of quinoline fluorescent probes and chemosensors, such combined probes—potentially allowing imaging—may be an interesting direction. Further chemical development should be compounded with a more robust application of computational methods, as seen in the field of photoswitches. A possible recent approach in the field of PPGs is the development of tandem systems combining 2PA antennae and PPG moieties [144], which have not yet been studied in depth for quinoline PPGs. From the structure‐property relationship studies, BHQ and CyHQ have emerged as two of the best performing 2PE PPGs so far, which are complemented by various DMAQ derivatives. In the present review, we have provided a comprehensive overview of the chemical evolution of quinoline PPGs, their mode of operation and characterization, and most importantly, their potential applications in biological/pharmacological experiments. The conclusions from the theoretical and SAR studies may contribute to the development of further probes and the identification of substrate classes not yet addressed by PPG‐based approaches. In this context, an interesting option is to use quinoline PPGs as photoactivatable building blocks for the development of photoresponsive nanoparticles or theranostic modalities. Photoactivatable nanoparticles allow the photorelease of more drug molecules at once, shifting advantageously the classical 1:1 PPG‐drug ratio. Previous reports on quinoline PPGs have mainly focused on theoretical and chemical aspects of PPG development. To fully exploit the potential of quinoline PPGs, further preclinical and in vivo characterization of PPG constructs with the joint effort of (medicinal) chemists and biologists/pharmacologists is required.

## Conflicts of Interest

The authors declare no conflicts of interest.

## Data Availability

The authors have nothing to report.
